# *Hyoscyamus albus* nortropane alkaloids reduce hyperglycemia and hyperinsulinemia induced in HepG2 cells through the regulation of *SIRT1*/*NF-kB/JNK* pathway

**DOI:** 10.1186/s12964-021-00735-w

**Published:** 2021-05-25

**Authors:** Anna Kowalczuk, Nabila Bourebaba, Katarzyna Kornicka-Garbowska, Eliza Turlej, Krzysztof Marycz, Lynda Bourebaba

**Affiliations:** 1 National Medicines Institute, Chełmska 30/34, 00-725 Warsaw, Poland; 2 International Institute of Translational Medicine, Jesionowa 11, 55-114 Malin, Wisznia Mała, Poland; 3 Department of Experimental Biology, Faculty of Biology and Animal Science, Wrocław University of Environmental and Life Sciences, Norwida 27B, 50-375 Wrocław, Poland; 4 Collegium Medicum, Institute of Medical Science, Cardinal Stefan Wyszyński University (UKSW), Dewajtis 5, 01-815 Warsaw, Poland

**Keywords:** *Hyoscyamus albus*, Calystegines, Insulin Resistance, Hyperglycemia, HepG2, *Sirt1*, *NF-κB*

## Abstract

**Background:**

Chronic superphysiological glucose and insulin concentrations are known to trigger several tissue and organ failures, including insulin resistance, oxidative stress and chronic low-grade inflammation. Hence, the screening for molecules that may counteract such conditions is essential in current existing therapeutic strategies, thereby the use of medicinal plant derivatives represents a promising axis in this regard.

**Methods:**

In this study, the effect of a selected traditional medicinal plant, *Hyoscyamus albus* from which, calystegines have been isolated, was investigated in an experimental model of hyperinsulinemia and hyperglycemia induced on HepG2 cells. The mRNA and protein expression levels of different insulin signaling, gluconeogenic and inflammatory pathway- related molecules were examined. Additionally, cell viability and apoptosis, oxidative stress extent and mitochondrial dysfunctions were assayed using flow cytometric and qRT-PCR techniques.

**Results:**

Treatment of IR HepG2 cells with calystegines strongly protected the injured cells from apoptosis, oxidative stress and mitochondrial integrity loss. Interestingly, nortropane alkaloids efficiently regulated the impaired glucose metabolism in IR HepG2 cells, through the stimulation of glucose uptake and the modulation of *SIRT1/Foxo1/G6PC/mTOR* pathway, which is governing the hepatic gluconeogenesis. Furthermore, the alkaloidal extract restored the defective insulin signaling pathway, mainly by promoting the expression of *Insr* at the mRNA and protein levels. What is more, treated cells exhibited significant mitigated inflammatory response, as evidenced by the modulation and the regulation of the *NF- κB/JNK/TLR4* axis and the downstream proinflammatory cytokines recruitment.

**Conclusion:**

Overall, the present investigation demonstrates that calystegines from Hyoscyamus albus provide cytoprotection to the HepG2 cells against insulin/glucose induced insulin resistance and apoptosis due to the regulation of *SIRT1/Foxo1/G6PC/mTOR* and *NF-κB/JNK/TLR4* signaling pathways.

**Video Abstract**

**Supplementary Information:**

The online version contains supplementary material available at 10.1186/s12964-021-00735-w.

## Background

Hyperinsulinemia (HI) and hyperglycemia are recognized as risk factors for a variety of abnormalities and clinical syndromes including type 2 diabetes and cardiovascular disease [1]. These diseases are often associated with hypertension, dyslipidemia and obesity suggesting the similar pathogenicity in the course of metabolic disorders [2]. Although not all obese patients are insulin resistant, the risk for insulin resistant development seems to directly correlate with increasing overweight/obesity [3]. As a consequence of sedentary life style among population all over the world, reduced physical activity and diet enriched with carbohydrates, especially in well developed countries, prevalence of metabolic disorders is increasing rapidly.

Hyperinsulinemia has been shown to play a crucial role in the course of hypertension, cardiovascular disease and as well as in liver metabolism leading to the development of non-alcoholic fatty liver diseases (NAFLD) [4]. NAFLD is currently one of the most chronic liver disorders with prevalence of more than 75% in obese individuals and type 2 diabetics [5]. Disease is associated with a wide spectrum of conditions including lipid accumulation which leads to hepatosteatosis, nonalcoholic steatohepatitis, advanced fibrosis and cirrhosis. The liver deterioration under hyperinsulinemia and hyperglycemia triggers hepatocyte apoptosis through induction of pro-apoptotic genes expression together with modulation of inflammatory reaction [6]. Diabetes and metabolic disorders are closely linked with systemic and tissue specific low-grade inflammation. Glucotoxicity and lipotoxicicty triggers oxidative and endoplasmic reticulum stress which stimulate the synthesis of inflammatory factors, further reducing the sensitivity of insulin target cells towards insulin, forming a vicious circle and aggravate insulin resistance. The release of active interleukin-1β (IL-1β) amplifies inflammation and contributes to cellular impairment [7]. On the other hand, free fatty acids in combination with fetuin, induce inflammatory state by the activation of toll-like receptor (TLR) and nuclear factor-κB (NF-κB) further supporting the synthesis of pro-inflammatory cytokines like tumor necrosis factor (TNF), IL-1β, interleukin 8 (IL-8) and monocyte chemoattractant protein-1 (MCP-1) [8]. Increased inflammation, caused by abundant infiltration of macrophages or mononuclear cells is one of critical component in the course of NAFLD related liver injuries. Recent data clearly indicate, that macrophages play a central role in modulating and initiating hyperinsulinemic-related tissue inflammatory responses [9, 10]. Accumulation of liver specific macrophages leads to enhanced secretion of pro-inflammatory cytokines which impair physiological functions of the organ and triggers insulin resistance. It has been shown, that knockout of monocyte chemotaxic protein-1 (MCP-1) or its receptor CCR2 in insulin resistant mice impairs macrophage inflammation and improves insulin sensitivity [11]. Another study has shown, that macrophage-specific knockout of IκB kinase (IKK) together with c-Jun N-terminal kinase (JNK) and Cbl-associated protein (CAP) have protective effect in obesity induced inflammation mice [12–14]. Moreover, the activation of NF-kB/JNK axis is recognized as one of the most critical components that link the macrophage inflammatory responses and systemic insulin resistance [15]. It has been demonstrated, that JNK activation might be involved in the development of steatohepatitis in obese patients and its suppression attenuates hepatic steatosis and improves insulin sensitivity [16, 17]. JNK1 might be activated by several HI-associated factors including inflammation, diglycerides and oxidative stress contributing to insulin resistance development through direct phosphorylation of the IRS-1 protein. The abundant accumulation of oxidative stress factors including reactive oxygen species (ROS) or nitric oxide (NO) together with reduced superoxide dismutase (SOD) activity leads to impairment of mitochondrial metabolism and dynamics, that might become an integrative factor of steatosis [18, 19]. The insulin resistance and hepatitis were shown to negatively affect the mitochondrial network leading to advantage of fission over fusion process [20]. It has been reported, that exposition of hepatocytes to hyperinsulinemia suppresses mitochondrial production and function through the classical Akt-dependent insulin signaling pathway [21]. HI is also recognized as an antagonist of the cAMP/PKA-dependent signaling pathway, which stimulates mitochondrial biogenesis and at the same time inhibits transcription of PGC-1α which is a key stimulator of mitochondrial biogenesis. Since activation of SIRT1 can lead to improved glucose tolerance and insulin sensitivity it is speculated that SIRT1/NF-kB/JNK axis could play an important role in regulating hyperinsulinemia and hyperglycemic in inflamed hepatocytes [22, 23].

*Hyoscyamus albus L*. is a Mediteranean plant which belongs to Solanaceae family and is known for its health-beneficial effects. *Hyoscyamus albus L* is rich in tropane alkaloids, mainly hyoscyamine and scopolamine, which has anticholinergic, analgesic, antispasmodic and sedative properties [24]. Recently, a new group of polyhydroxylated nortropane alkaloides called calystegines have been found in *Hyoscyamus,* which sheds a promising light for its application for biomedical application [25, 26]. Calystegines as polyhydroxyalkaloids display glycosidase-inhibitory properties by mimicking the pyranosyl or furanosyl moiety of their natural substrates. Recently, it has been shown, that calystegines are potent, promising antidiabetic agents with antihyperglycemic and hypolipidemic effects. The streptozotocin induced diabetic mice treated with calystegines showed minimized streptozotocine damages on β-cells of islets of Langerhans, stimulated β-cells regeneration and improved with this insulin secretion [25].

In this study we’ve investigated whether *Hyoscyamus albus* nortropane alkaloids reduce hyperglycemia and hyperinsulinemia induced in HepG2 cells through the modulation of oxidative stress, the improvement of glucose metabolism and the regulation of SIRT1/NF-kB/JNK pathway.

## Materials and methods

### Plant sampling

*Hyoscyamus albus* seeds were collected from Tizi-Ouzou province (Bouzguene location) of Algeria with semi-arid climate. Samples were then cleaned by removing the dried calyxes, seeds were dehydrated during two weeks, and grounded in order to obtain a fine powder that was stored in the dark and dry conditions.

### Chemicals

Cell culture reagents were purchased from BioWest (VWR International, Gdańsk, Poland), and chemicals as well as reagents, unless otherwise mentioned, were obtained from Sigma Aldrich (Poznań, Poland).

### Total Calystegines isolation

To obtain total calystegines of *H. albus* powdered seeds, the extraction was accomplished according to the protocol of Bourebaba et al. [27]. 50 g of powder seeds were three times prior to hydroalcoholic extraction defatted with 250 ml petroleum ether. After that, crud extract was homogenizing with 250 ml aqueous methanol (50/50; v/v), three times each 24 h. Calystegines were separated from others components of the dried extract using a cation exchange column (Amberlite IR 120B, H+ form). Consequently, all contaminants were eliminated from the column using distilled water, and the linked compounds were eluted with NH_4_OH (2 N). An anion exchange column (Dowex 1X2, Cl− form) was subsequently used in order to elude calystegine-rich fraction from concentrated residue with distilled H_2_O.

### Gas chromatography–mass spectrometry analysis (GC–MS)

According to the method defined by Bourebaba et al. [27], total calystegine extract was characterized by gas chromatography–mass spectrometry analysis. First, a step of trimethylsilyl trifluoroacetamide (MSTFA) derivatization was performed preceding chromatographic analysis, and then, a GCMS-QP2010 plus system (Shimadzu, Kyoto, Japan) prepared with a DB-5 ms column (30 m × 0.25 mm I.D. × 0.25 μm df, Quadrex Corporation, Woodbridge, CT) was engaged for the characterization. The extract compounds separation was carried out in accordance with the following temperature program: initial temperature of 100 °C kept for 5 min, then increased to 300 °C at 10 °C/min, sustained during 5 min. The injection volume in split mode (split ratio 1:10) was 0.5 µl with the injector temperature at 250 °C, and the gaz carrier was He at 36.5 cm/s. interface temperature, 280 °C; ion source temperature, 250 °C; mass range, m/z 50–600; scan speed, 2500 amu/seg; and event time, 0.20 seg., were the MS detection parameters employed for the experiment. Using the GCMS solution (ver. 2.50SU3, Shimadzu) program, data collection and handling was carried out.

### Cell culture

Human hepatocellular carcinoma (HepG2) cell line was purchased from ATCC (American Type Culture Collection, Rockville, MD, USA) and maintained at the Department of Experimental Biology at the Wroclaw University of Environmental and Life Science in Poland. HepG2 cell line was cultured in low-glucose DMEM medium (Dulbecco’s modified Eagle’s medium) supplemented with 10% heat inactivated FBS (fetal bovine serum), 2 mM L-glutamine and the solution of penicillin–streptomycin 100x. Cells were cultured at 37 °C, in 5% CO_2_, and humid atmosphere till they reached 70–80% of confluence.

### Biocompatibility assessment of Hyoscyamus albus total calystegines

The cytocompatibility of the isolated nortropane alkaloids was evaluated in terms of cell growth, viability and apoptosis. HepG2 cells were seeded in 96-well plates for cytotoxicity assay, and in 6-well plates for cell viability and apoptosis assessment, and left to attach in culture incubator for 24 h. Calystegines (10–500 μg/ml) were thereafter supplemented to the cultures at different concentrations and incubated for 24 h and 48 h for cytotoxicity assay, and tested at 250 µg/ml for 24 h for cell viability and apoptosis evaluation in normal conditions. Growth rate was then estimated using the resazurin-based (TOX-8) kit test solution; and cellular viability was investigated by the means of a Muse Annexin V & Cell Death kit (Cat. N° MCH100105), as well as by the analysis of apoptotic-related genes expression. Resazurin reduction was measured using a spectrometer (BMG Labtech, Germany) for microplates at a specific 600 nm wavelengths and 690 nm reference wavelengths, and results were normalized to a negative control. Total living and apoptotic cells were monitored using the Muse Cell analyzer. Relative expression of apoptotic genes was determined using qRT-PCR technique.

### Experimental model of insulin resistance establishment and related treatments

Insulin resistance model was established by exposing cells to hyperglycemia and hyperinsulinemia condition, which mimics type 2 diabetic model and its associated insulin resistance condition. Briefly, HepG2 cells were seeded onto culture dishes, and kept in standard culture conditions for cellular attachment. Afterwards, cells were pre-treated with the single predefined optimal calystegines concentration, i.e. 250 µg/ml for 24 h. All experimental groups were subsequently exposed to high D-glucose (30 mM) and high insulin (500 nM) in serum-free medium for another 24 h after an over-night serum-starving [28]. A group of untreated healthy HepG2 cells was included and cultured under the same experiment conditions to serve as a control group.

### Cytotoxicity in vitro test

The HepG2 cell line was seeded on the 96-well plastic plates in triplicates at the density of 1 × 10^4^ cells per well in DMEM complete medium in the final volume of 100 µl and cultured for 24 h and then for next 24 h with studied calystegines. The measurement of the toxic effect of high D-glucose and high insulin (30 mM/500 nM) toward the HepG2 cell line was based on the metabolic activity of living cells. The cell growth rate was evaluated using in vitro TOX-8 assay kit from Sigma-Aldrich, Poznań, Poland according to the manufacturer’s protocol. Briefly, after incubation time, the used medium above the cells was replaced by complete medium with the addition of 10% resazurin dye solution and the plates were incubated at 37 °C, in 5% CO_2_, and humid atmosphere for next 2 hs. The absorbance was read using Elisa plate reader (BioTek, Epoch, Swindon, UK) equipped with Gen5 software at 600 nm as the primary wavelength and 620 nm as a reference wavelength. The results are presented in the form of mean values ± SD. Each experiment was repeated 3 times.

### Flow cytometry analysis of apoptotic cells

The evaluation of the percentage of the apoptotic cells in samples was performed using Annexin-V and Dead Cell Assay Kit from Merck Millipore, Darmstadt, Germany according to supplier’s protocol. Briefly, cells were seeded on the 6-well plastic plates in the complete DMEM and were incubated at 37 °C, in 5% CO_2_, in the presence or not of the related treatments. After incubation time, cells were collected using Trypsin–EDTA 1 × in PBS without calcium, magnesium and phenol red, washed with HBSS and centrifuged at 324 g, 5 min, 4 °C and suspended in HBSS with addition of the 1% BSA. The Muse Annexin V and Dead Cell Reagent was added to the cell’s suspension in ratio 1:1 (v/v) and after 20 min incubation at RT in the dark, cells were read out using Muse™ Cell Analyzer equipped with Muse™ Software. The percentage of apoptotic cells was calculated with an assumption that double negative cells are viable cells, only annexin V positive are early apoptotic cells, only 7-AAD positive are necrotic cells and finally double positive cells are late apoptotic cells. Each experiment was performed independently 3 times.

### Determination of multicaspase activity

The evaluation of the number and percentage of cells with positive caspase activity (1,3,4,5,6,7,8 and 9) was performed using Muse™ Multicaspase Kit from Merck Millipore, Darmstadt, Germany according to supplier’s protocol. Briefly, cells were seeded on the 6-well plastic plates in the complete DMEM and were incubated at 37 °C, in 5% CO_2_, and humid atmosphere for 24 hs with 250 µg/ml calystegines and then for next 24 hs with glucose/insulin combination (30 mM/500 nM). After incubation time, cells were collected using Trypsin–EDTA 1 × in PBS without calcium, magnesium and phenol red, washed with HBSS and centrifuged at 324 g, 5 min, 4 °C, suspended in MultiCaspase Reagent working solution in PBS (1:160) and incubated for 30 min in the 37 °C with 5% CO_2_. After incubation time, Muse™ Caspase 7-AAD working solution in 1 × Caspase Buffer (1:74) was added to each sample and after 5 min incubation at RT in the dark, cells were read out using Muse™ Cell Analyzer equipped with Muse™ Software. The percentage of pan caspase positive cells were calculated with an assumption that double negative cells are viable cells, only caspase positive are pan caspase positive cells, only 7-AAD positive are necrotic cells and finally double positive are cells in late stages of the caspase activity. Each experiment was performed independently 3 times.

### Analysis of mitochondrial transmembrane potential

The mitochondrial dysfunction associated with the ability of high glucose and high insulin for depolarization of the inner membrane mitochondrial potential (ΔΨ) was evaluated using Muse™ MitoPotential Kit from Merck Millipore, Darmstadt, Germany according to supplier’s protocol. Briefly, cells were seeded on the 6-well plastic plates in the complete DMEM and incubated at the at 37 °C, in 5% CO_2_, and humid atmosphere for 24 hs under the same previously detailed experimental conditions. After incubation time, cells were collected using Trypsin–EDTA 1 × in PBS without calcium, magnesium and phenol red, washed with HBSS and centrifuged at 324 g, 5 min, 4 °C and suspended at the density 0.5 × 10^6^ cells/ml in 1 × Assay Buffer. Next, MitoPotential working solution in 1 × Assay Buffer (1:1000) was added to the cells and suspension were incubated for 20 min in the 37 °C with 5% CO_2_. After incubation time, Muse MitoPotential 7-AAD reagent was added to the cell suspension for 5 min and the cells were read out using Muse™ Cell Analyzer equipped with Muse™ Software. The total percentage of cells with depolarized mitochondrial membrane was assessed with an assumption that the cells positive only on MitoPotential axis are viable with intact mitochondrial potential, cells positive for 7-AAD are dead with intact mitochondrial potential, and the sum of double positive cells (dead cells with depolarized mitochondrial membrane) and double negative (viable cells with depolarized mitochondrial membrane) reflects total percentage of cells with mitochondrial dysfunction. Each experiment was performed independently 3 times.

Mitochondria were additionally stained using the MitoRed fluorescent dye. Following each related treatment, the cells were incubated with MitoRed solution (dilution 1:1000) at 37 °C for a period of 30 min. Cells were afterwards washed three times with HBSS and subsequently fixed with 4% PFA at room temperature for 40 min. The cells were counterstained with 4’,6-diamidino-2-phenylindole (DAPI) contained in ProLong Gold Antifade mounting medium (Life Technologies, Warsaw, Poland), in order to visualize the cells’ nuclei. All groups of cells were visualized using a confocal microscope (Observer Z1 Confocal Spinning Disc V.2 Zeiss with live imaging chamber). Photomicrographs were captured with a Canon PowerShot camera and processed using the ImageJ software. Estimated fluorescence intensity of MitoRed was presented as the pseudo-ratio (∆F/Fo), following the previously described method of Quintanilla et al. [29].

### Flow cytometry analysis of ROS overproduction

The evaluation of the number and the percentage of cells undergoing oxidative stress was performed using Muse Oxidative Stress Kit from Merck Millipore, Darmstadt, Germany according to supplier’s protocol. Briefly, cells were seeded on the 6-well plastic plates in the complete DMEM and received the different established treatments. After incubation time, cells were collected using Trypsin–EDTA 1 × in PBS without calcium, magnesium and phenol red, washed with HBSS and centrifuged at 324 g, 5 min, 4^0^C and suspended at the density of 1 × 10^6^/ml in 1 × Assay Buffer. The Muse Oxidative Stress Reagent working solution in 1 × Assay Buffer (1:79) was added to each sample, cells were incubated for 30 min at 37^0^C and read out using Muse™ Cell Analyzer equipped with Muse™ Software. The percentage of cells in M2 gate reflects cells exhibiting ROS. Each experiment was performed independently 3 times.

### Glucose uptake assay

To examine the glucose uptake, the fluorescent 2-NBDG glucose analogue was used. A treatment of HepG2 cells with total *H. albus* calystegine extract for 24 h, followed by 30 mM/500 nM D-glucose/insulin exposure for an additional 24 h was performed. Cells were washed for three times with PBS and then incubated with 2-NBDG (100 µM) for another 30 min at 37 °C. To stop the 2-NBDG uptake reaction, the remaining culture medium was removed, and subsequently cells were washed with cold HBSS, and fixed for 15 min at room temperature with 4% PFA. HepG2 cells were labelled with DAPI (Life Technologies, Warsaw, Poland) contained in ProLong Gold Antifade and were visualized by confocal (Observer Z1 Confocal Spinning Disc V.2 Zeiss with live imaging chamber). Relative corrected fluorescent intensities have been obtained using the ImageJ software and represented as the 2-NBDG/DAPI ratio.

### Real Time Reverse Transcription analysis of genes expression

The expression of genes associated with apoptosis, insulin signaling, gluconeogenesis, oxidative stress, mitochondrial dysfunction and inflammation summarized in Table 1, was evaluated from samples secured in TRIzol according to manufacturer’s recommendation. The cells were seeded on the 6-well plastic plates and incubated with calystegines prior to IR induction. After incubation time, cells were collected using Trypsin–EDTA 1 × in PBS without calcium, magnesium and phenol red, washed with HBSS and centrifuged at 324 g, 5 min, 4 °C and secured in TRIzol. Quantity and purity of isolated RNA was assessed using spectrophotometer at 260 nm wavelength (WPA, Biowave II, Biochrom, Cambridge, UK). 150 ng RNA was used to cDNA synthesis using Tetro cDNA Strand cDNA Synthesis Kit (Bioline, London, UK) based on oligo (dT) primers in T100 Thermal Cycler (BioRad, Hercules, CA, USA). The detection of target gene expression was performed using a SensiFAST SYBR Green Kit (Bioline, London, UK) in CFX Connect™ Real-Time PCR Detection System (BioRad, Hercules, CA, USA). The relative expression has been taken to GAPDH (glyceraldehydes-3-phosphate, housekeeping gene) expression.

**Table 1:** Sequences of primers used in qPCR

Gene	Primer	Sequence 5'–3'	Amplicon length (bp)	Accession No
*Mfn1*	F: R:	GTTGCCGGGTGATAGTTGGA TGCCACCTTCATGTGTCTCC	146	NM_033540.3
*Mfn2*	F: R:	AATCTGAGGCGACTGGTGAC GGACATTGCGCTTCACCTTC	126	XM_024451299.1
*Parkin*	F: R:	GTGCAGAGACCGTGGAGAAA GCTGCACTGTACCCTGAGTT	294	NM_013987.3
*Sod1 (cu/zn sod)*	F: R:	CATTCCATCATTGGCCGCAC GAGCGATCCCAATCACACCA	130	NW_001867397.1
*Sod2 (mn sod)*	F: R:	GGACAAACCTGAGCCCCAAT TTGGACACCAGCCGATACAG	125	NW_001867408.1
*Cat*	F: R:	ACCAAGGTTTGGCCTCACAA TTGGGTCAAAGGCCAACTGT	112	XM_014851065.1
*GPx*	F: R:	TCCGGGACTACACCCAGATG TCTTGGCGTTCTCCTGATGC	108	NM_000581.4
*TLR4*	F: R:	GACGGTGATAGCGAGCCAC TTAGGAACCACCTCCACGCAG	173	NM_138554.5
*Pink1*	F: R:	GCTTGGGACCTCTCTTGGAT CGAAGCCATCTTGAACACAA	142	NM_032409.3
*NF-kB-p65*	F: R:	CTGTTCCCCCTCATCTTCCC GTATCTGTGCTCCTCTCGCC	113	L19067.1
*c-Jun*	F: R:	ACGGCGGTAAAGACCAGAAG CCAAGTTCAACAACCGGTGC	89	NM_002228.4
*IL-1β*	F: R:	AAACAGATGAAGTGCTCCTTCCAG TGGAGAACACCACTTGTTGCTCCA	391	NM_000576.3
*IL-4*	F: R:	CTTTGCTGCCTCCAAGAACAC GCGAGTGTCCTTCTCATGGT	97	NM_000589.4
*IL-6*	F: R:	TCCTTCTCCACAAACATGTAACAA ATTTGTGGTTGGGTCAGGGG	319	NM_001318095.2
*TNF-α*	F: R:	AGTGACAAGCCTGTAGCCCA GTCTGGTAGGAGACGGCGAT	242	NM_000594.4
*p53*	F: R:	AGATAGCGATGGTCTGGC TTGGGCAGTGCTCGCTTAGT	381	NM_001126118.1
*Insr*	F: R:	TAGACGTCCCGTCAAATATTGC GAAGAAGCGTAAAGCGGTCC	244	AH002851.2
*Irs2*	F: R:	GAGCTGTGGCGTTTCACATC AGCTTCGGGCTGAAACAGT	234	NM_003749.3
*Akt1*	F: R:	CTGTCATCGAACGCACCT GTCTGGATGGCGGTTGTC	178	NM_005163.2
*Akt2*	F: R:	TCAAAGAAGGCTGGCTCCAC TGTACCCAATGAAGGAGCCG	205	M95936.1
*Pi3k*	F: R:	TTTAATCTGCCAGGCGGAGG CCAGAATTCCATGGGGCAGT	151	NM_006218.4
*Bax*	F: R:	ACCAAGAAGCTGAGCGAGTGTC ACAAAGATGGTCACGGTCTGCC	356	XM_011527191.1
*Bcl-2*	F: R:	ATCGCCCTGTGGATGACTGAG CAGCCAGGAGAAATCAAACAGAGG	129	NM_000633.2
*p21*	F: R:	AGAAGAGGCTGGTGGCTATTT CCCGCCATTAGCGCATCAC	169	NM_001220777.1
*Casp3*	F: R:	CTCTGGTTTTCGGTGGGTGT CTTCCATGTATGATCTTTGGTTCC	136	NM_004346.4
*Casp9*	F: R:	CAGGCCCCATATGATCGAGG CTGGCCTGTGTCCTCTAAGC	142	NM_032996.3
*Sirt1*	F: R:	TCACTGCACCACTTGAGTCC ACAGGTTGCGGGAATCCAAA	73	NM_012238.5
*Foxo1*	F: R:	CTCTGGCCCCTTTCACCATA GCTTTCTTCTTGGCAGCTCG	229	XM_011535010.2
*G6PC*	F: R:	CACTTCCGTGCCCCTGATAA AGTATACACCTGCTGTGCCC	95	NM_000151.4
*GAPDH*	F: R:	GTCAGTGGTGGACCTGACCT CACCACCCTGTTGCTGTAGC	256	NM_001289746.1

*Mfn1*, Mitofusin 1; *Mfn2*, Mitofusin 2; *Parkin*, Parkin RBR E3 ubiquitin protein ligase (PARK2); *Pink1*, PTEN-induced putative kinase 1; *Sod1 (Cu/Zn SOD)*, Copper-zinc-dependant superoxide dismutase *(CuZnSOD*; *Sod2 (Mn SOD)*, Manganese-dependent superoxide dismutase (MnSOD*)*; *CAT*, *Catalase*; *GPx*, *Glutathione Peroxidase*; *TLR4*, Toll like receptor 4; *NF-kB-p65*, Nuclear Factor-kappa-B transcription factor p65; *c-Jun*, Jun Proto-Oncogene, AP-1 Transcription Factor Subunit; *IL-1β*, Interleukin 1 beta; *IL-4*, Interleukin 4; *IL-6*, Interleukin 6; *TNF-α*, Tumor Necrosis Factor alpha; *P53*, tumor suppressor p53; *Insr*, Insulin Receptor; *Irs2*, Insulin Receptor Substrate 2; *Akt1*, Serine/threonine 308 Kinase 1; *Akt2*, serine/threonine kinase 2; *Pi3k*, Phosphoinositide 3-Kinase; *Bcl-2*, B-cell lymphoma 2; *Bax*, BCl-2 associated X protein; *p21*, Cyclin-dependent kinase inhibitor 1; *Casp3*, Caspase 3; *Casp9*, Caspase 9; *Sirt1*, Sirtuin 1; *Foxo1*, forkhead box O1; *G6PC*, Glucose 6 phosphate Catalytic subunit; *GADPH*, Glyceraldehyde-3-phosphate dehydrogenase

### Western immunoblot analysis

HepG2 cells were collected from each experimental culture dishes and homogenized in lysis buffer (Tris at 50 mmol/L pH 7.4, NaCl at 150 mmol/L, SDS 0.1%, sodium deoxycholate 0.5%, protease cocktail, 1 mmol/L PMSF, 10 mmol/L of sodium ascorbate, 1% Triton X-100, 10 mmol/L sodium azide, and Trolox at 5 mmol/L), in addition to phosphatase and protease inhibitor mixture on ice, in order to analyze the proteins profiles. Supernatants were transferred to fresh tubes after centrifugation for 20 min at 4 °C and 6000 × g, to remove the insoluble materials, and processed at − 80 °C until further use. The protein concentration was determined using the Pierce™ Bicinchoninic Acid (BCA) Protein Assay Kit (Life Technologies, USA), and cell lysates were initially diluted with 4 × Laemmli loading buffer (Bio-Rad, USA), subsequently denatured at 65 °C for 10 min in line to proteins separation. Samples were subjected to SDS–polyacrylamide gel electrophoresis in Tris/glycine/SDS buffer 100 V using Mini-PROTEAN Tetra Vertical Electrophoresis Cell (Bio-Rad, USA) during 90 min, and transferred to the polyvinylidene difluoride (PVDF) membranes (Bio-Rad, USA) with Mini Trans-Blot®Cell (Bio-Rad, USA) transfer apparatus in Tris/glycine buffer/methanol with 100 V, 250 mA at 4 °C for 45 min. Non-phosphorylated proteins membranes were blocked in 5% non-fat milk solution in TBST, and phosphorylated proteins membranes were blocked in 5% BSA in TBST. Proteins were detected with primary antibodies (Table 2) and HRP-conjugated secondary antibodies (dilution 1:2500 in TBST, 1 h incubation at room temperature) by incubation overnight at 4 °C. Chemiluminescent signals were screened and quantified with Image Lab Software (Bio-Rad, USA), using the ChemiDoc MP Imaging System (Bio-Rad, USA).

**Table 2:** List of antibodies employed for proteins profiling using western blot analysis

Antibody	Dilution	Catalog No
*Insr*	1:1000	Invitrogen, MA1-10865
*Akt Pan*	1:1000	Invitrogen, 44-609G
*Pi3kCD*	1:1000	Invitrogen, PA5-83748
*SIRT1- N-terminal region*	1:1000	ARP32386
*TOR/mTOR*	1:500	nb100-240
*NF-kappaB p65(phospho-T254)*	1:1000	Biorbyt, orb304547
*JNK1/JNK2(phospho-Thr183/Tyr185)*	1:1000	Biorbyt, orb15028
*Actin*	1:2000	Sigma Aldrich, a2066

*Insr*, Insulin Receptor; *Akt*, Protein Kinase B; *Pi3kCD*, Phosphatidylinositol 3-Kinase; *SIRT1*, Sirtuin 1; *TOR/wTOR*, Target of rapamycin/Mammalian Target of rapamycin; *NF-kappaB p65(phospho-T254)*, Phosphorylated Nuclear Factor-kappa-B transcription factor p65; *JNK1/JNK2(phospho-Thr183/Tyr185)*, Phosphorylated c-Jun N-terminal kinase

### Statistics

All statistical analyses were performed using GraphPad Prism 5.0 (San Diego, CA, USA) with a one-way analysis of variance (ANOVA) followed by Bonferroni’s post-hoc multiple comparison test, as indicated. Asterisk (*) and Hash (#) signs indicate statistical significance in the HI/HG-induced groups versus the healthy control or in the HI/HG-induced control versus the Calystegines-treated groups, respectively. All p values lower than 0.05 (*p* < 0.05) are summarized with one asterisk/hash (*/#), those at *p* < 0.01 use two asterisks/hashes (**/##), and those at *p* < 0.001 have three asterisks/hashes (***/###).

## Results

### Calystegines content in *Hyoscyamus albus* seeds extract

**Fig. 1:**
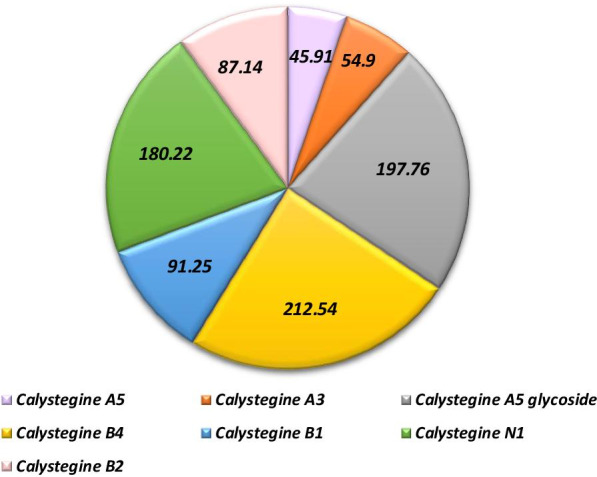
GC–MS phytochemical screening of *Hyoscyamus albus* calystegines isolated from seeds. Concentrations are given as μg calystegines/g DW of seeds. Identification and analysis were based on the monitoring of the following ions in a scanning mod: 217 m/z: calystegines B; 229 m/z: calystegine B2; 156 m/z: calystegines A; 390 m/z: calystegine N1; 375 m/z: calystegine C1; 189 m/z: calystegine B1; 71 m/z: octadecane; 1: calystegine A5; 2: calystégine A3; 3: calystegineA5 Gly.; 4: calystegine B4; 5: calystegine B1; 6: calystegine N1; 7: calystegine B2 [27, 29]

Based on the previous analysis of *Hyoscyamus albus* seeds total calystegines using GC–MS [27], Fig. 1 summarises the different identified calystegines as well as their content in the studied extract. The phytochemical screening demonstrated the presence of three A group calystegines (A3, A5, and A5 glycoside) as well as calystegine N1 in the seeds extract, in appreciable amounts. Moreover, the analysis brought out the co-isolation of three B group calystegines (B1, B2, and B4), calystegine B4 being the most profuse along all the present nortropanes [27].

### In vitro cytocompatibility of nortropane alkaloids on Hepatocarcinoma Cells

**Fig. 2:**
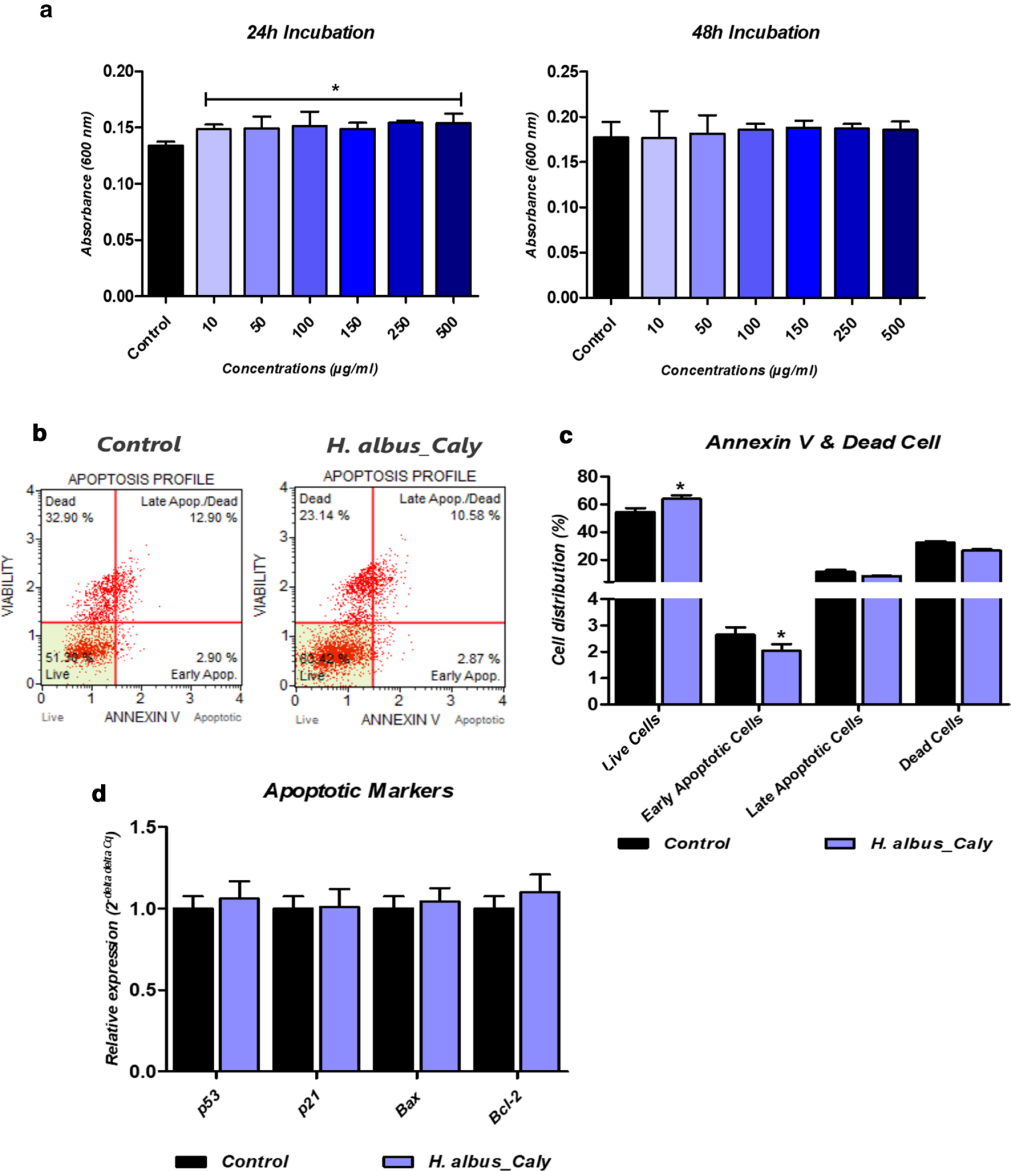
Effect on cell proliferation, viability and apoptosis of *H. albus* calystegines on HepG2 cells. **a** Histograms represent the average absorbance at 600 nm of the Alamar Blue assay. **b** Representative dot-plots for Annexin V & Dead Cell assay **c** Bar-charts depicting the quantitative analysis of live, early, and late apoptosis and cell death. **d** Relative gene expression of main apoptotic markers normalized to GAPDH housekeeping gene. Representative data from three independent experiments are shown ± SD (n = 3). An asterisk (*) indicates a comparison of treated group to untreated healthy cells. * *p* < 0.05, ** *p* < 0.01, *** *p* < 0.001. Control: HepG2 healthy untreated cells; *H. albus*_Caly: HepG2 cells treated with 250 μg/ml of the total calystegines extracted from *Hyoscyamus albus* seeds for 24 h

To investigate the effect of total calystegines isolated from *H. albus* seeds, on the cell viability of human hepatocarcinoma cell line, a resazurin-based assay, an Annexin V & Dead Cell test as well as an analysis of apoptotic markers related genes expression were performed. The cells were cultured in the presence of various concentrations (10–500 μg/ml) of calystegines during 24 and 48 h. Obtained results showed no deleterious effect on the cellular viability of HepG2 cells after 24 h and 48 h incubation respectively (Fig. 2a). The cell survival rate was above 90% on all concentrations even at a highest concentration of 500 μg/ml, as compared to control cells. Interestingly, calystegines exhibited a relative stimulatory effect on cells proliferation after 24 h incubation (*p* < 0.05), but not after 48 h incubation (Fig. 2a). Annexin V & Dead Cell assay similarly resulted in an enhancement of total living cells percentage after calystegines supplementation at a final concentration of 250 µg/ml, as compared to healthy untreated cells (Fig. 2c). Moreover, no changes in the total apoptotic and dead cells levels were observed upon 24 h calystegines treatment respectively. Analysis of gene expression of apoptosis regulators confirmed the non-influence of nortropane alkaloids on cellular apoptosis; as no significant differences in both *p53*, *p21* and *Bax* transcripts levels were monitored between treated and untreated HepG2 cells (Fig. 2d).

### Calystegines protect from HI/HG induced cell death in HepG2 cells

**Fig. 3:**
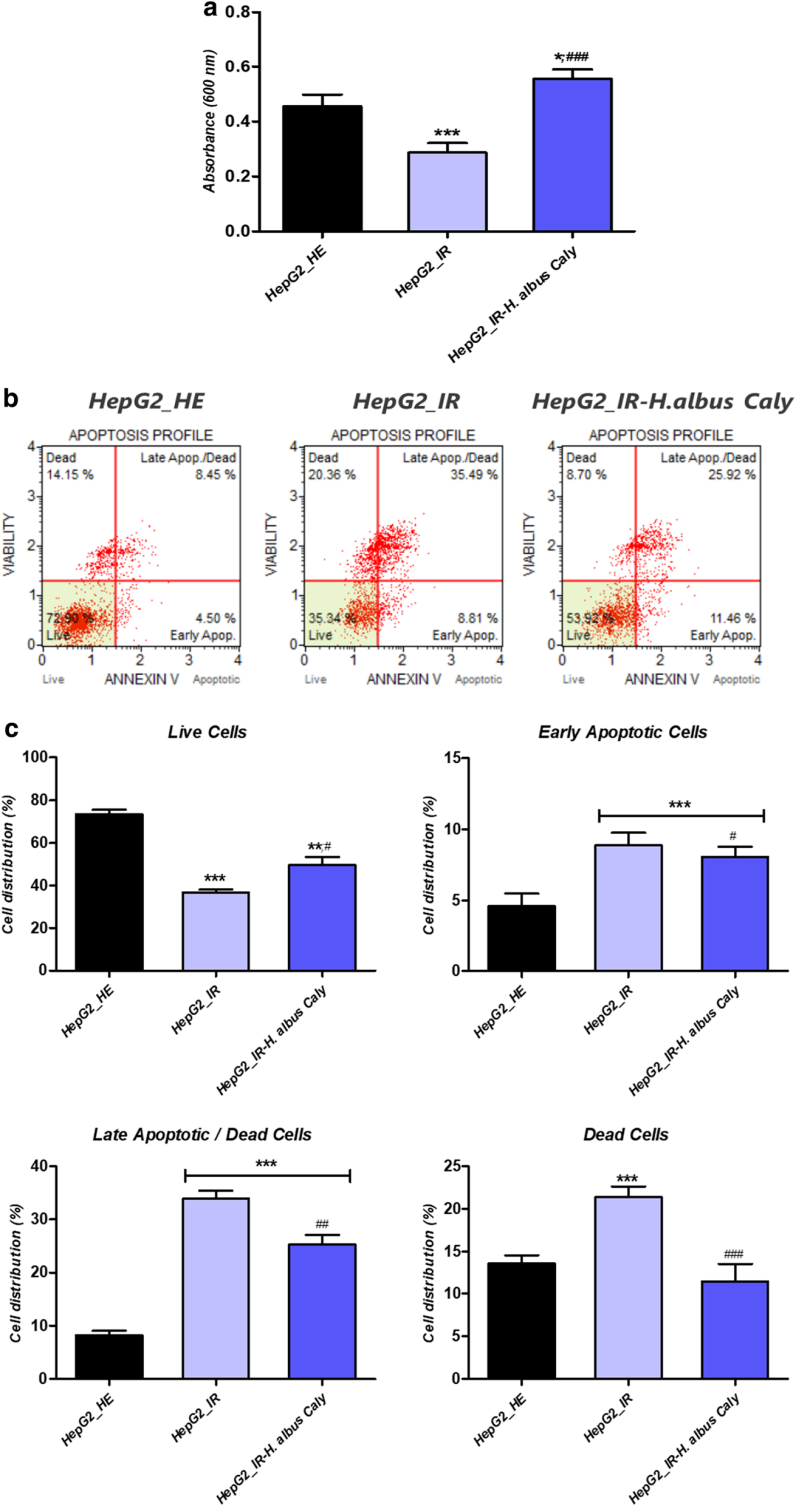

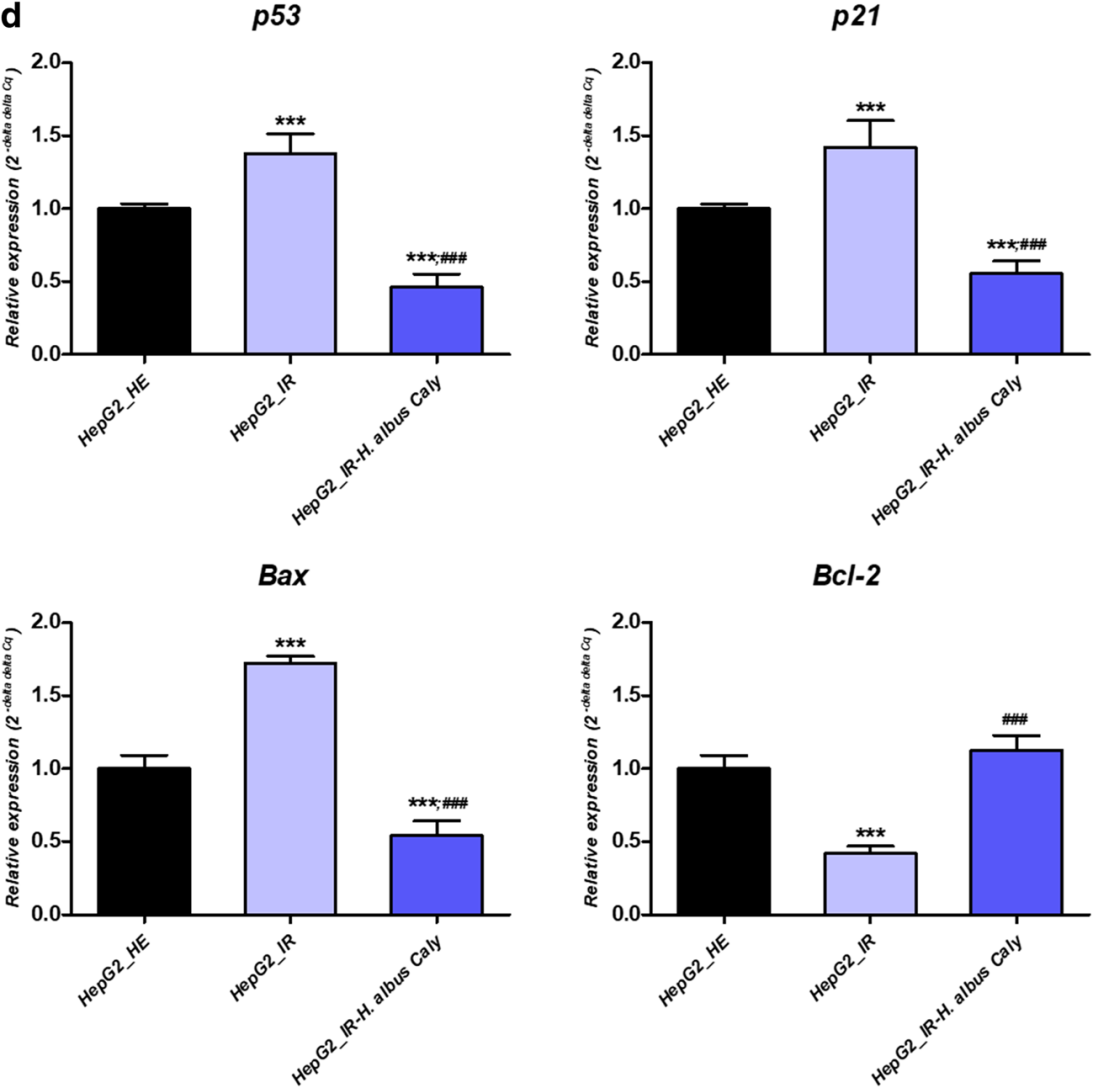
Influence of total calystegines pre-treatment on HepG2 cells viability and apoptosis challenged by prolonged exposure to high insulin and glucose levels. **a** Cell proliferation assessed by the Tox8 assay. **b** Representative apoptosis profile plots from flow cytometry analysis. **c** Quantitative estimation of Annexin V/7-AAD positive and negative cells. **d** Relative expression quantitation of main apoptosis-associated markers levels. Representative data from three independent experiments are shown ± SD (n = 3). An asterisk (*) indicates a comparison of IR group to untreated healthy cells. A hashtag (#) indicates a comparison of IR group pre-treated with calystegines to IR untreated healthy cells. */#*p* < 0.05, **/##*p* < 0.01, ***/###*p* < 0.001. HepG2_HE: HepG2 healthy untreated cells; HepG2_IR: Insulin resistant HepG2 cells exposed to high concentrations of insulin and glucose. HepG2_IR-*H. albus*_Caly: Insulin resistant HepG2 cells exposed to high concentrations of insulin and glucose and pre-treated with 250 μg/ml calystegines extracted from *Hyoscyamus albus* seeds

To explore whether the application of notropane alkaloids may reduce the induced apoptotic cell death related to hyperinsulinemia (HI) and hyperglycaemia (HG) condition in HepG2 cells, Tox8 test, Annexin V & Dead Cell assay as well as gene expression analysis of pro-apoptotic factors have been carried out for the investigation of apoptotic cell death in cultured cells. As demonstrated in Fig. 3a, incubation of HepG2 cells in hyperglycaemic and hyperinsulinemic microenvironment for 24 h resulted in a considerable reduction of the metabolically active cells rate, which corresponds to a reduction in cellular viability and proliferation as compared to untreated healthy cells (*p* < 0.001). As similarly observed with biocompatibility results, calystegines at a final concentration of 250 µg/ml significantly improved cellular viability, and stimulated proliferation of HepG2 cells (*p* < 0.05; *p* < 0.001), in comparison to both healthy and pathologic groups of cells (Fig. 3a). Likewise, flow cytometric analysis revealed a higher percentage of cells exhibiting a loss of membrane integrity and highly expressed phosphatidylserine under HI/HG condition, which is associated with increased apoptotic events (Fig. 3c), by contrast to healthy control (*p* < 0.001). Supplementation of cultures with nortropane alkaloids resulted in a sharp mitigation of apoptosis upon IR induction; as evidenced by the decreased number of Annexin V-positive cells at late apoptosis stage, as well as a moderation of the total amount of dead cells when compared to both healthy and IR cells (*p* < 0.05; *p* < 0.001). At the same time, prolonged simultaneous exposure of cells to high insulin and glucose doses produced a remarkable overexpression of pro-apoptotic regulators *p53*, *p21* and *Bax* transcripts (Fig. 3d), while strongly suppressing the expression of *Bcl-2* mRNA in regards to healthy hepatocytes (*p* < 0.001). Pre-treatment of experimental cultures led to a positive regulation of above-mentioned transcripts levels, when compared to IR cells (*p* < 0.001). More interesting, the alkaloidal extract significantly downregulated the relative levels of *p53*, *p21* and *Bax* below the basal threshold of healthy HepG2 cells (*p* < 0.001). Consequently, calystegines have been found to additionally promote the transcription of the pro-survival *Bcl-2* gene by way of contrast to IR control (*p* < 0.001).

### Calystegines prevent the activation of caspases triggered by HI/HG in HepG2 cells

**Fig. 4:**
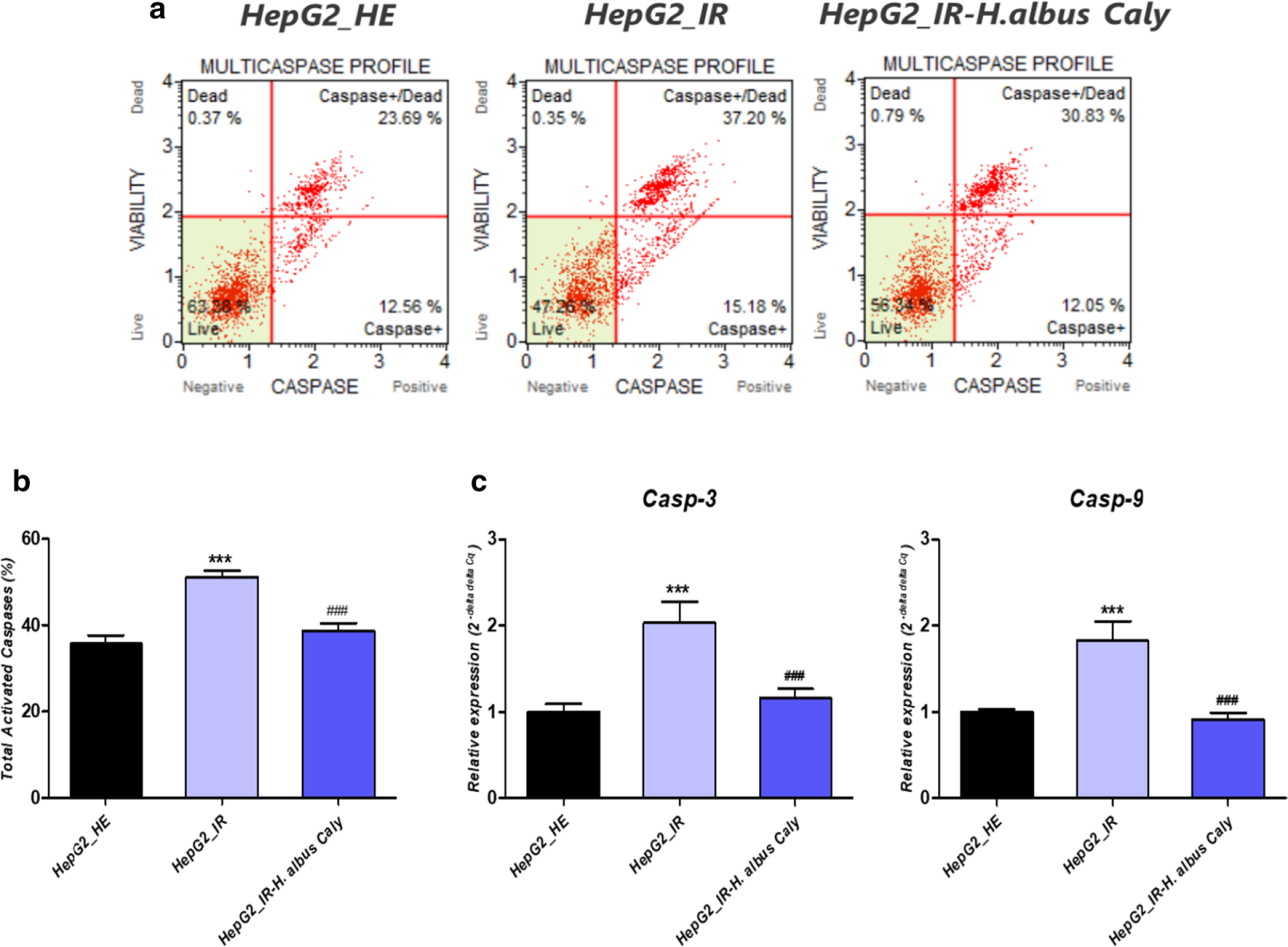
Impact of calystegines preconditioning on caspases expression and activation over HI/HG induction of HepG2 cells. **a** Representative plots from the Muse™ MultiCaspase Assay. **b** Bar charts depict the percentage of total cells with multicaspase enzyme activation. **c** mRNA expression of caspase‐3 and caspase-9 assayed by quantitative real‐time polymerase chain reaction (PCR). Representative data from three independent experiments are shown ± SD (n = 3). An asterisk (*) indicates a comparison of IR group to untreated healthy cells. A hashtag (#) indicates a comparison of IR group pre-treated with calystegines to IR untreated healthy cells. */#*p* < 0.05, **/##*p* < 0.01, ***/###*p* < 0.001. HepG2_HE: HepG2 healthy untreated cells; HepG2_IR: Insulin resistant HepG2 cells exposed to high concentrations of insulin and glucose. HepG2_IR-*H. albus*_Caly: Insulin resistant HepG2 cells exposed to high concentrations of insulin and glucose and pre-treated with 250 μg/ml calystegines extracted from *Hyoscyamus albus*

To further elucidate the extend of total calystegines isolated from *H. albus* in counteracting apoptosis that results from HI/HG applied conditions, the activation as well as the expression patterns of caspases have been assessed through flow cytometry and RT-qPCR respectively. Muse Pan Caspase analysis evidenced a critical mobilization of cysteine proteases-1, 3, 4, 5, 6, 7, 8, and 9 in HepG2 cells following excessive insulin and glucose supplementation (Fig. 4b), as compared to HepG2 cells cultured under physiological conditions (*p* < 0.001). Moreover, insulin resistant cells displayed higher expression levels on both Casp-3 and Casp-9 transcripts (Fig. 4c). Interestingly, pre-treatment if cultured cells with nortropane alkaloids engendered a 1.33-folds dwindling of total activated caspases by contrast to IR control cells (*p* < 0.001), to achieve a rate comparable to the basal level exhibited by healthy cells (Fig. 4b). Similarly, the extract also allowed a suppression of the overexpression of the two genes coding for the effector caspases 3 and 9 (Fig. 4c).

### Calystegines ameliorate glucose metabolism in HI/HG challenged HepG2 cells

**Fig. 5:**
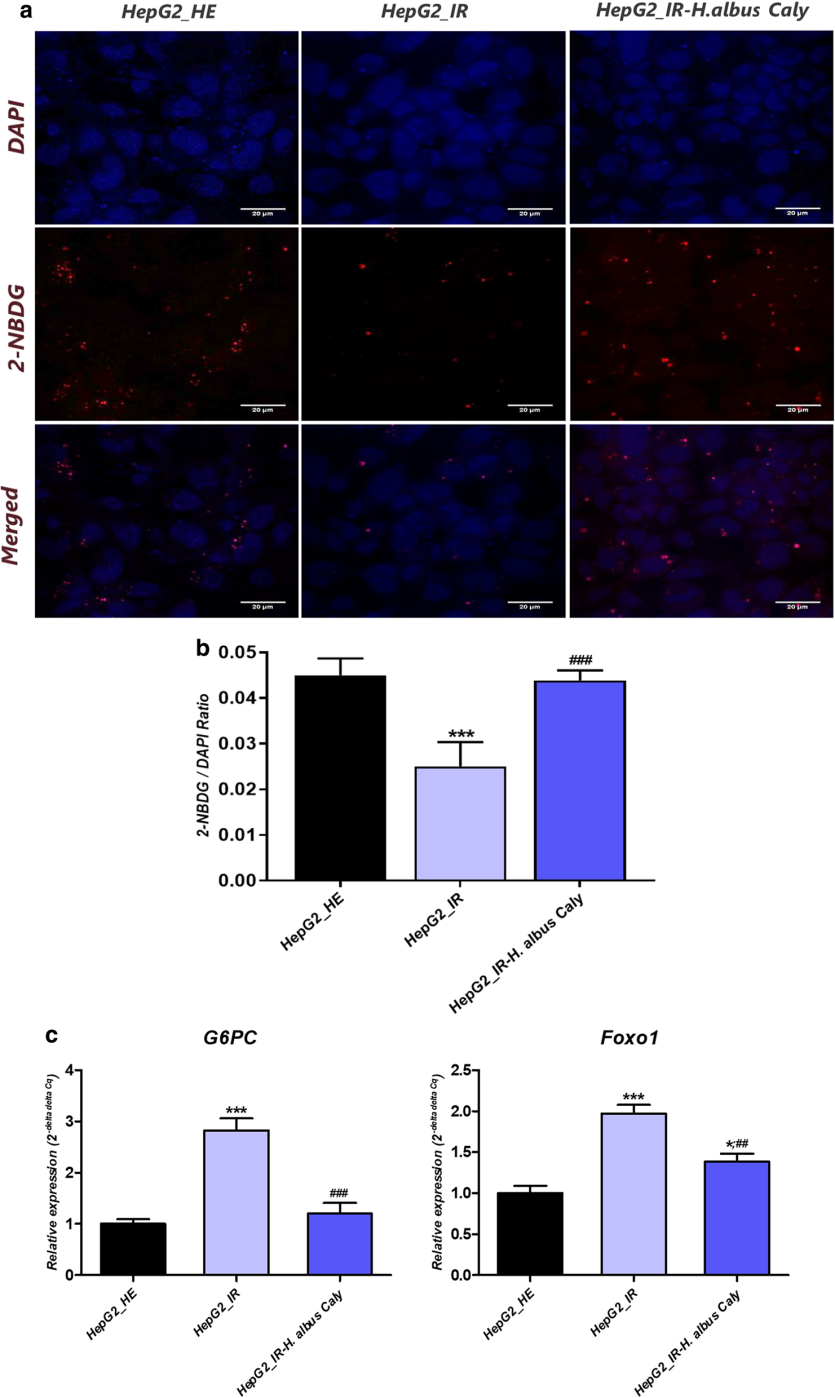
Effect of calystegines on glucose uptake and gluconeogenesis regulation in IR HepG2 cells. **a** Representative photomicrographs of 2-NBDG uptake assay obtained by confocal epi-fluorescent microscopy; Bar size 20 μm; magnification × 60. **b** 2-NBDG/DAPI relative fluorescence ratio quantification. **c** Expression of gluconeogenic genes evaluated by qRT-PCR. Representative data from three independent experiments are shown ± SD (n = 3). An asterisk (*) indicates a comparison of IR group to untreated healthy cells. A hashtag (#) indicates a comparison of IR group pre-treated with calystegines to IR untreated healthy cells. */# *p* < 0.05, **/## *p* < 0.01, ***/### *p* < 0.001. HepG2_HE: HepG2 healthy untreated cells; HepG2_IR: Insulin resistant HepG2 cells exposed to high concentrations of insulin and glucose. HepG2_IR-*H. albus*_Caly: Insulin resistant HepG2 cells exposed to high concentrations of insulin and glucose and pre-treated with 250 μg/ml calystegines extracted from *Hyoscyamus albus* seeds

The potential role of nortropane alkaloids on the glucose uptake and glucose metabolism in insulin-resistant HepG2 cells was evaluated using fluorescent detection and transcriptomic analysis. As represented in Fig. 5a photomicrographs, stimulation of HepG2 cells with high concentrations of insulin and glucose for 24 h significantly reduced the absorptive capacities of cells regarding 2-NBDG as evidenced by the diminished red fluorescent signal in comparison to healthy hepatocytes. By contrast, hepatocarcinoma cells pre-treated with 250 µg/ml calystegines displayed obvious and visible stronger fluorescent signal emanating from the fluorescent glucose analogue 2-NBDG absorbed in greater quantity than in IR cells (Fig. 5b). To further elucidate the influence of calystegins on hepatic glucose production, which is known to be responsible for the exacerbation of glucotoxicity, relative mRNA expression of key gluconeogenesis-related genes, namely glucose-6-phosphatase catalytic subunit (G6PC), and forkhead box protein O1 (Foxo1) was screened. Prolonged hyperglycemia and hyperinsulinemia-developed insulin resistance in HepG2 cells strongly triggered the overexpression of *G6PC* and *Foxo1*, by opposition to healthy cells (*p* < 0.001), denoting the excessive gluconeogenesis within IR hepatocytes, and the resulting de novo synthesis of glucose, which has been identified as one of the major causes of hyperglycaemia in the course of type 2 diabetes. The mRNA levels of the same genes were however markedly decreased in the calystegines 250 µg/ml group compared with the IR group, showing a potential regulatory effect on endogenous de novo glucose production (Fig. 5c).

### Calystegines improve glucose metabolism through the regulation of the impaired SIRT1/mTOR pathway in IG HepG2 cells

**Fig. 6:**
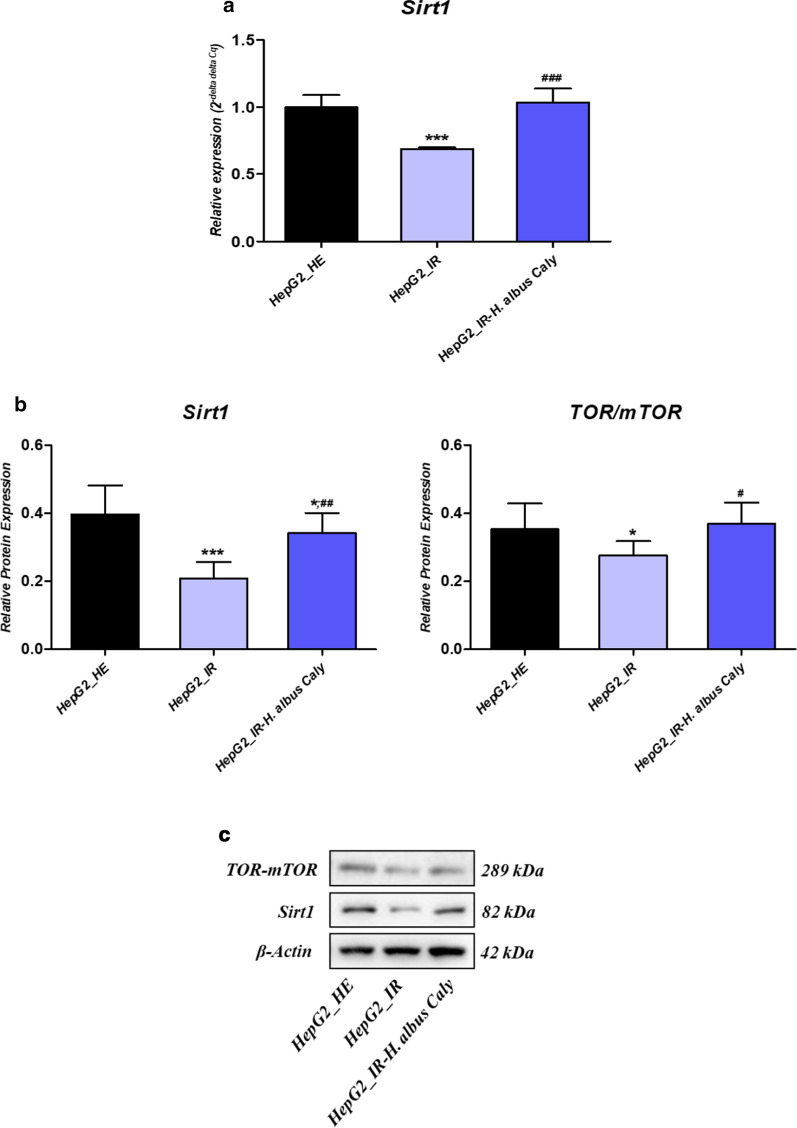
regulatory potential of calystegines on Sirt/mTOR axis in the course of IR induced in HepG2 cells. **a** Average relative expression of Sirt1 gene normalized to houskeeping GAPDH gene. **b** Relative quantitative abundance of Sirt1 and mTOR proteins obtained from western blot analysis. **c** Representative immunoblots for each assayed protein detected by chemiluminescence. Representative data from three independent experiments are shown ± SD (n = 3). An asterisk (*) indicates a comparison of IR group to untreated healthy cells. A hashtag (#) indicates a comparison of IR group pre-treated with calystegines to IR untreated healthy cells. */#*p* < 0.05, **/##*p* < 0.01, ***/###*p* < 0.001. HepG2_HE: HepG2 healthy untreated cells; HepG2_IR: Insulin resistant HepG2 cells exposed to high concentrations of insulin and glucose. HepG2_IR-*H. albus*_Caly: Insulin resistant HepG2 cells exposed to high concentrations of insulin and glucose and pre-treated with 250 μg/ml calystegines extracted from *Hyoscyamus albus* seeds

To elucidate whether the normalization of glucose metabolism associated with *Foxo1* and *G6PC* expression under calystegines treatment may be mediated by an eventual effect on upstream modulators, the analysis of the expression patterns of *Sirt1*, an NAD+-dependent deacetylase, that is a key regulator of glucose and lipid homeostasis controlling *Foxo1* and *GP6C* activation, as well as other downstream effectors such as *mTOR*, involved in insulin pathway modulation has been considered in the present study. The induction of an insulin resistance state using high doses of insulin and glucose on human hepatocytes resulted in a considerable collapse of the expression level of the gene encoding *Sirt1* (Fig. 6a), compared to physiologically healthy cells (*p* < 0.001). As a consequence, the relative abundance of the Sirt1 protein (Fig. 6b) was significantly below the basal expression level of the healthy cells under the same conditions (*p* < 0.001). It similarly followed that the expression of *TOR*/*mTOR* protein has been strongly suppressed in the same group of cells (*p* < 0.001), indicating an alteration of the *Sirt1*/*mTOR* axis regulating carbohydrates homeostasis. Here, the pre-treatment of experimental cells with a single dose of calystegines efficiently restored the expression of both *Sirt1* and *mTOR* at both protein and gene levels by opposition ti the IR untreated group (*p* < 0.01; *p* < 0.05), thus revealing that nortropanic alkaloids act on glucose metabolism through the regulation and enhancement of the *Sirt1*/*Foxo1*/*mTOR* signalling cascades.

### Calystegines enhance insulin signalling afflicted by HI/HG condition in HepG2 cells

**Fig. 7:**
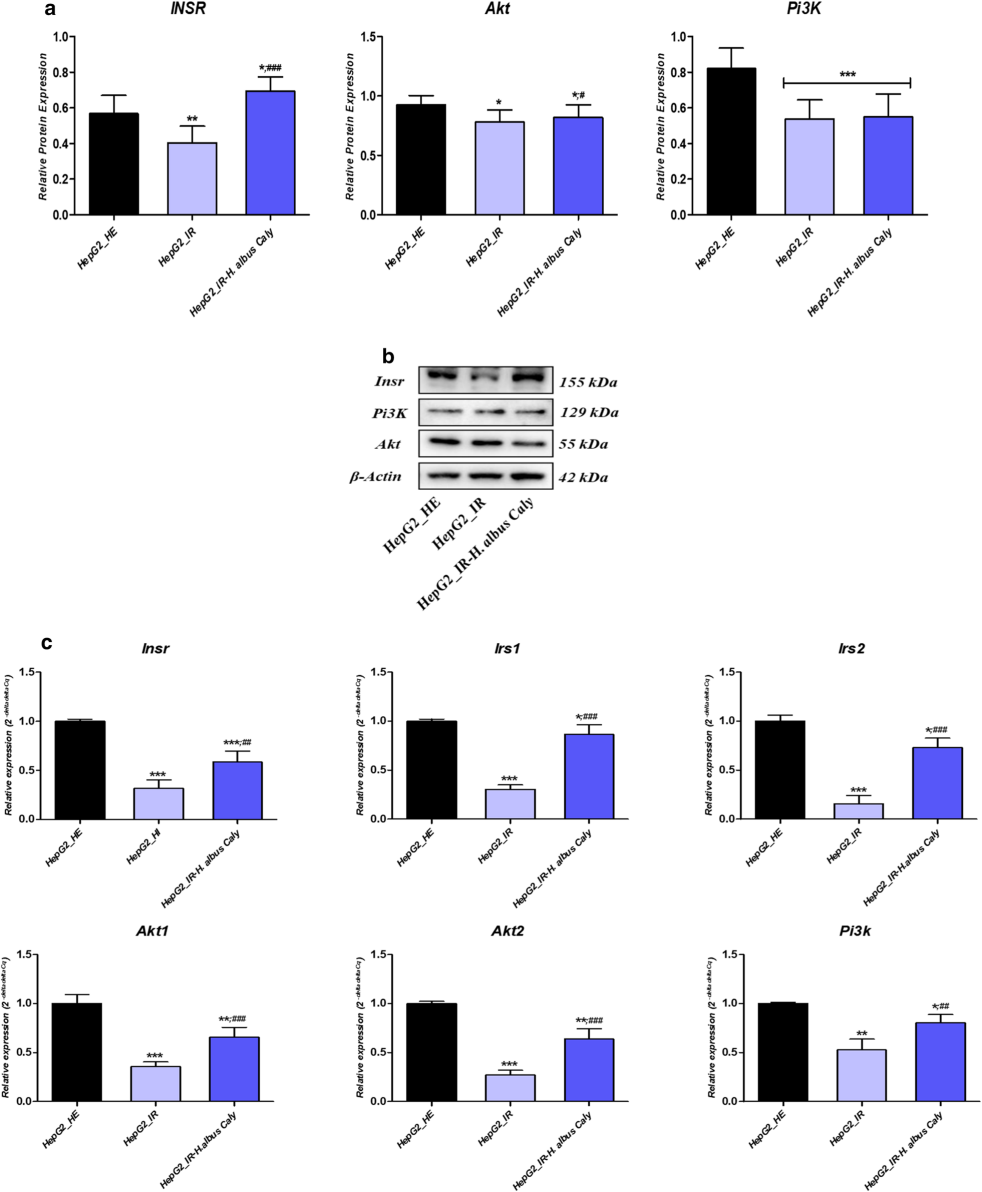
Effect of total calystegines on collapsed insulin signalling pathway in HI/HG HepG2 cells. **a** Quantitative analysis of insulin signalling-related proteins expression using western blot. **b** Representative blots images of insulin signalling-related proteins profiling. **c** Relative genes expression of key insulin signalling regulators. Representative data from three independent experiments are shown ± SD (n = 3). An asterisk (*) indicates a comparison of IR group to untreated healthy cells. A hashtag (#) indicates a comparison of IR group pre-treated with calystegines to IR untreated healthy cells. */#*p* < 0.05, **/##*p* < 0.01, ***/###*p* < 0.001. HepG2_HE: HepG2 healthy untreated cells; HepG2_IR: Insulin resistant HepG2 cells exposed to high concentrations of insulin and glucose. HepG2_IR-*H. albus*_Caly: Insulin resistant HepG2 cells exposed to high concentrations of insulin and glucose and pre-treated with 250 μg/ml calystegines extracted from *Hyoscyamus albus* seeds

To determine if the observed effect on glucose uptake and metabolism is mediated by the regulation of insulin signalling related pathway, the activation of *Insr*, *Akt* and *Pi3k* was assayed after calystegines pre-treatment. As shown in Fig. 7a, exposure of HepG2 cells to high levels of insulin and glucose resulted in a decrease in *Insr*, *Akt* and *Pi3k* proteins expression, by opposition du healthy cells (*p* < 0.01; *p* < 0.05; *p* < 0.001), which is a salient feature of hyperglycaemic and hyperinsulinemic liver cells. Nortropan alkaloids supplementation triggered a moderated restoration of *Akt* protein expression but did not affect the expression patterns of *Pi3k* protein. What is more interesting, calystegines significantly up-regulated the relative expression of *Insr* protein not only in regards to the untreated pathological group, but also compared to the group of healthy cells (*p* < 0.001; *p* < 0.05). Expression of insulin related effectors has also been evaluated at the transcriptional level. Obtained data (Fig. 7c) clearly demonstrated a marked suppression of all *Insr*, *Akt1*, *Akt2*, *Irs1*, *Irs2* and *Pi3k* transcripts in IR HepG2 cells (*p* < 0.001); which were subsequently up-regulated in the HepG2 cultures that were preincubated with 250 µg/ml calystegines for 24 h prior to HI/HG induction, in relation to untreated group (*p* < 0.001), reporting on the regulatory potential of calystegines on insulin signalling pathway activation.

### Calystegines modulate oxidative stress and mitochondrial dysfunction associated with HI/HG condition in HepG2 cells

**Fig. 8:**
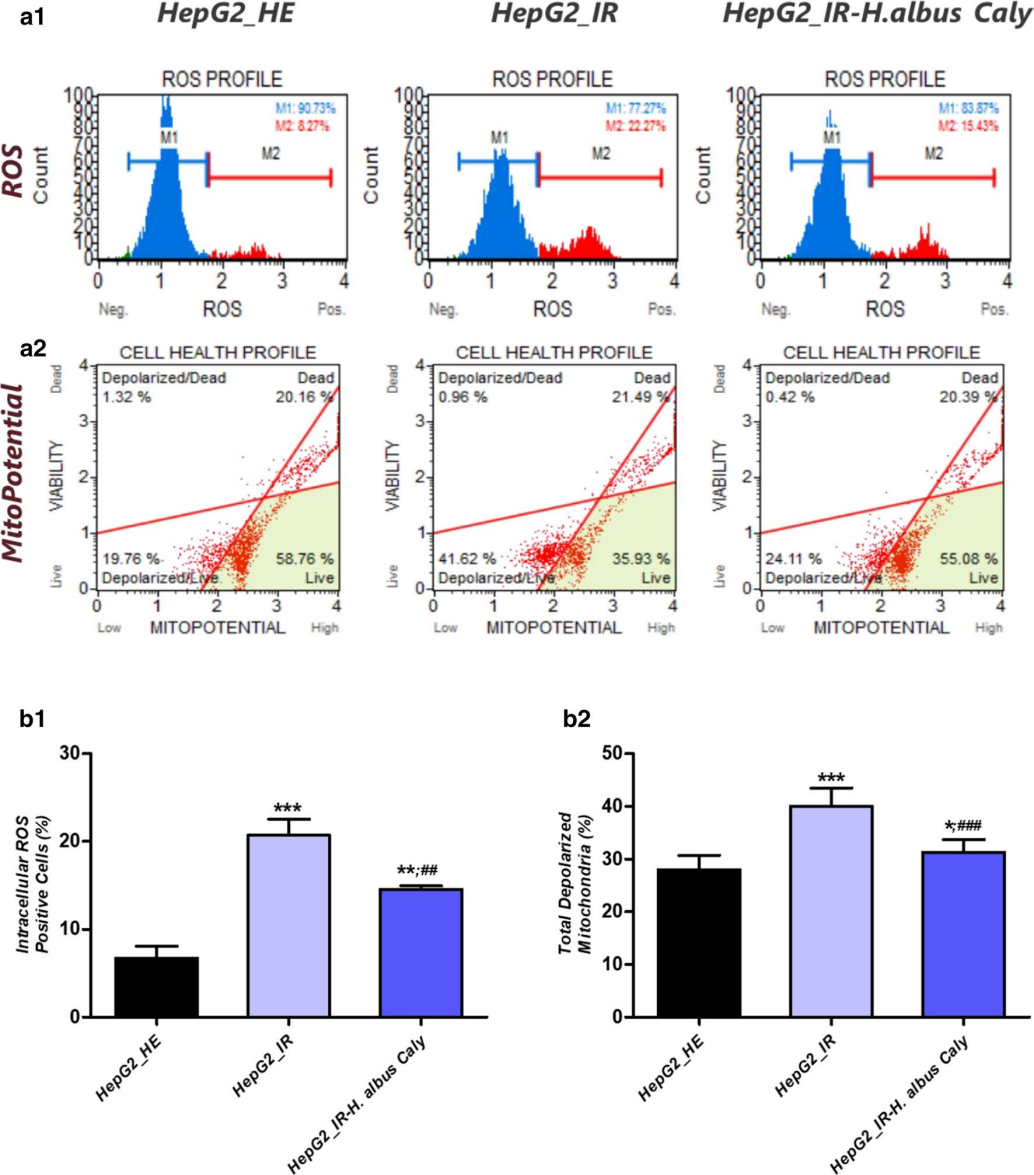

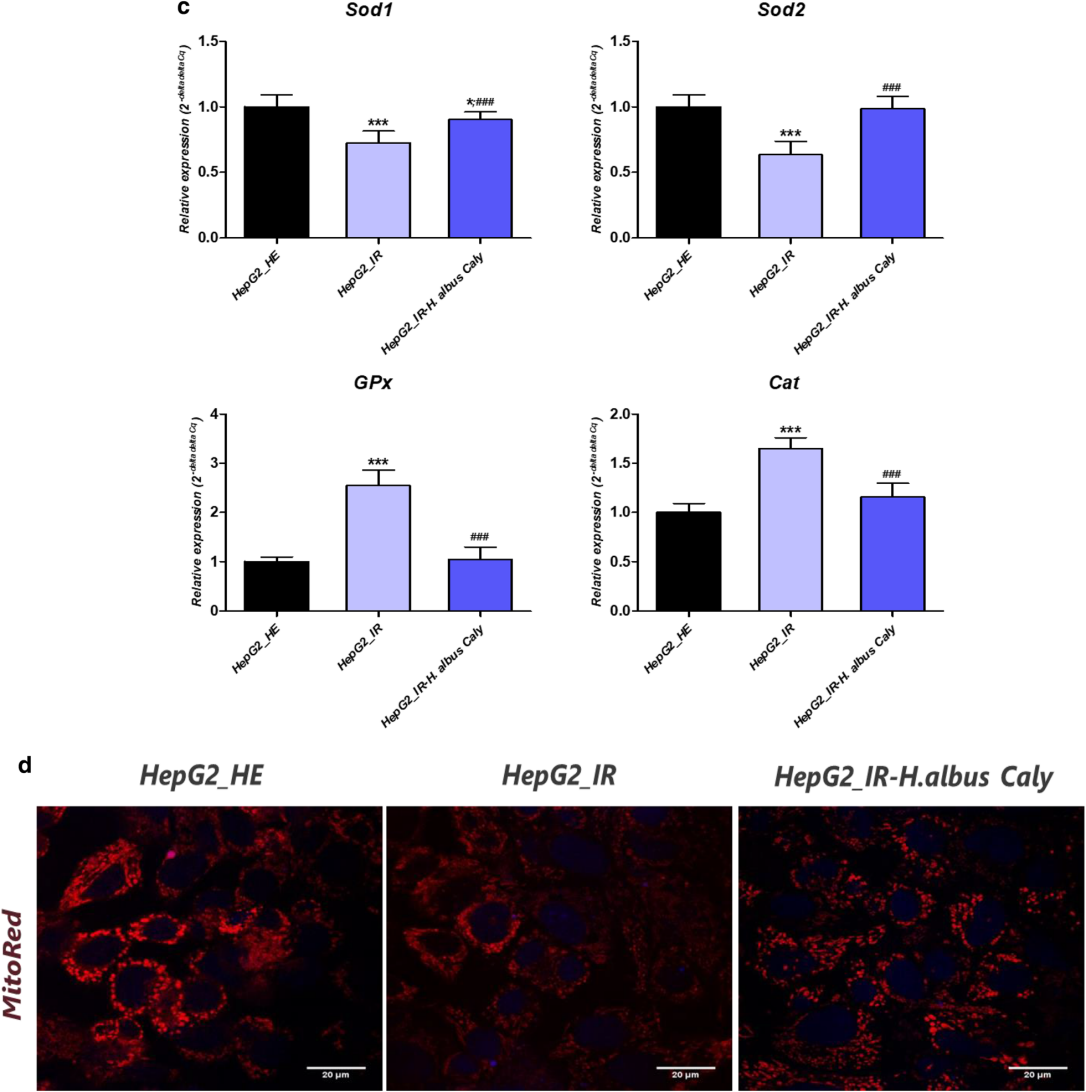

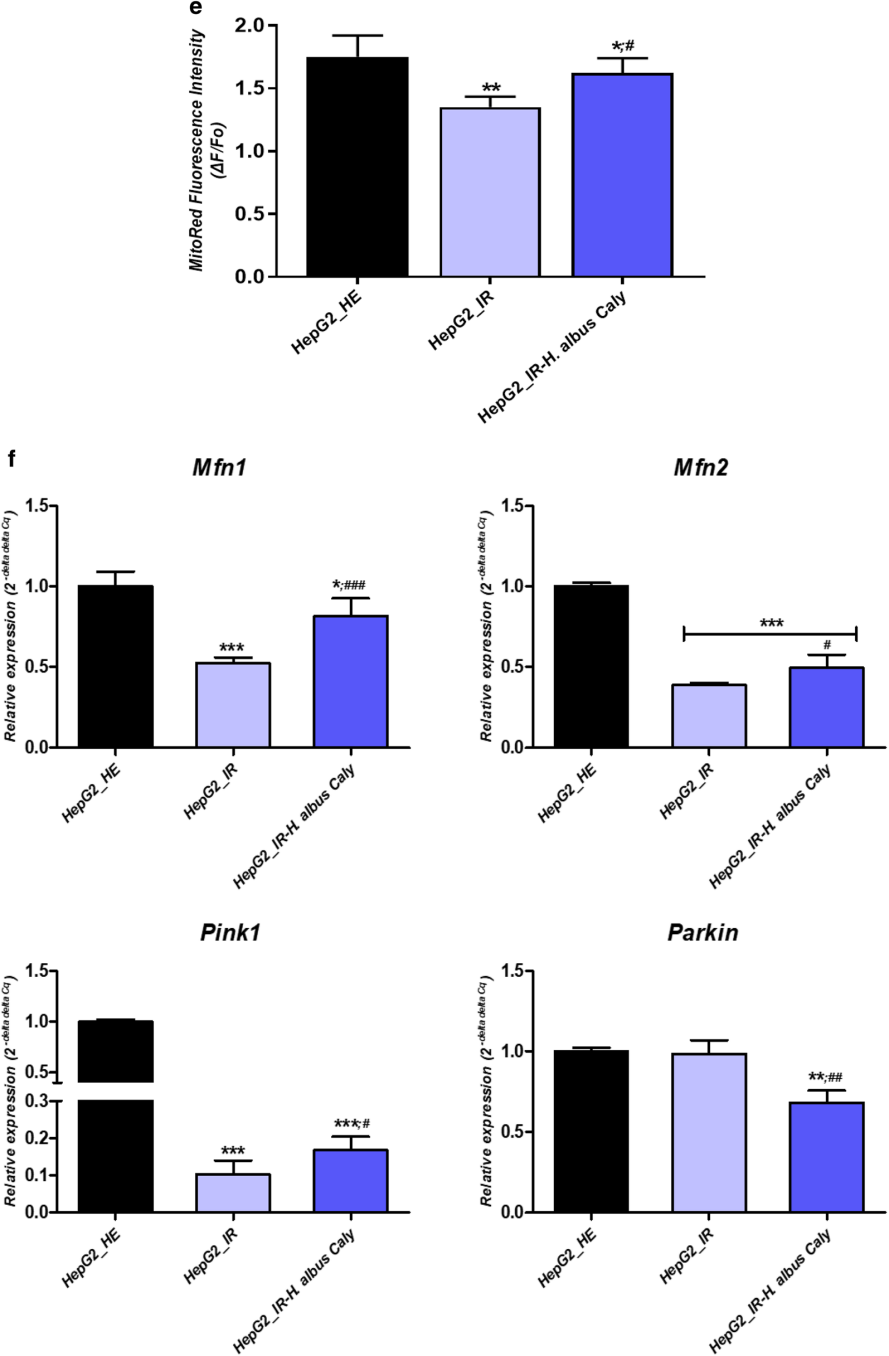
Impact of calystegines treatment on oxidative stress and mitochondrial dysfunction in HI/HG challenged HepG2 cells. **a1** Representative plots for events distribution from Muse Oxidative Stress Assay. **a2** Representative dot plots for Muse Mitopotential Assay. **b1** Level of intracellular ROS formation represented by the percentage of ROS positive cells. **b2** Histograms showing the average percentages of total cells exhibiting depolarized mitochondria. **c** Relative gene expression of *Sod1*, *Sod2*, *Cat* and *GPx* antioxidant enzymes transcripts. **d** Illustration of confocal micrographs for cells labelled with MitoRed fluorescent dye; scale bar size 20 μm; magnification set at 60-folds. **e** Quantification of MitoRed fluorescent intensities using the pseudo ratio ΔF/F0. **f** Bar-charts demonstration the levels of main mitochondrial dynamics-related mRNAs. Representative data from three independent experiments are shown ± SD (n = 3). An asterisk (*) indicates a comparison of IR group to untreated healthy cells. A hashtag (#) indicates a comparison of IR group pre-treated with calystegines to IR untreated healthy cells. */#*p* < 0.05, **/##*p* < 0.01, ***/###*p* < 0.001. HepG2_HE: HepG2 healthy untreated cells; HepG2_IR: Insulin resistant HepG2 cells exposed to high concentrations of insulin and glucose. HepG2_IR-*H. albus*_Caly: Insulin resistant HepG2 cells exposed to high concentrations of insulin and glucose and pre-treated with 250 μg/ml calystegines extracted from *Hyoscyamus albus* seeds

Glucotoxicity and hyperinsulinemia are important factors that contribute to liver cells failure through the onset of persistent oxidative stress, that is believed as a key event in diabetic complications. In this study, high glucose and insulin treatment rapidly increased the levels of intracellular ROS in HepG2 cells in contrary to cells that received no supplementary insulin and glucose (Fig. 8b2). Additionally, the gene expression analysis of the four main important antioxidant enzymes showed a decline in *Sod1* and *Sod2* mRNA levels and a compensatory upregulation of *Cat* and *GPx* transcripts, as a consequence of ROS overproduction (Fig. 8c). Alkaloidal extract of *H. albus* applied 24 h prior to HI/HG induction protected HepG2 cells from oxidative stress development, as evidenced by the significant reduction in intercellular ROS (*p* < 0.001). Furthermore, the plant extract exerted a remarkable regulatory effect on the expression extend of the analysed antioxidant genes, probably mediated through the rehabilitation of the oxidative stress-induced imbalance. As one of the main molecular pathways involved in oxidative stress extension, lies in the mitochondrial electron transfer system impairment, HepG2 mitochondrial alterations were also monitored after the different related treatments. As expected, intoxicated cells exhibited elevated levels of depolarized mitochondria as compared to healthy control group (*p* < 0.01); while cells preconditioned with calystegines presented significant diminished percentage of total depolarized mitochondria (Fig. 8b2). The visualization of stained mitochondrial net under confocal microscope similarly demonstrated a much more homogeneous and well-defined mitochondrial network in groups treated with calystegines, with a higher fluorescent signal, suggesting a stronger mitochondrial biogenesis occurrence (Fig. 8d, e). In addition, exposure of hepatocytes to a microenvironment overdosed in insulin and glucose, strongly altered the mitochondrial dynamic pathways (Fig. 8f) via the downregulation of the *Pink1*, *Mfn1* and *Mfn2* encoding genes, when compared to normal cells (*p* < 0.001), while no changes in the expression of *Parkin* mRNA were observed in the same group. Likewise, total alkaloids application occasioned a noticeable regulation of altered transcripts (Fig. 8f), whereas it interestingly decreased the transcription of *Parkin* mRNA by regards to both control groups (*p* < 0.01).

### Calystegines imped the Insulin resistance activated NF-κB/JNK and inflammatory cytokines in HepG2 cells

**Fig. 9:**
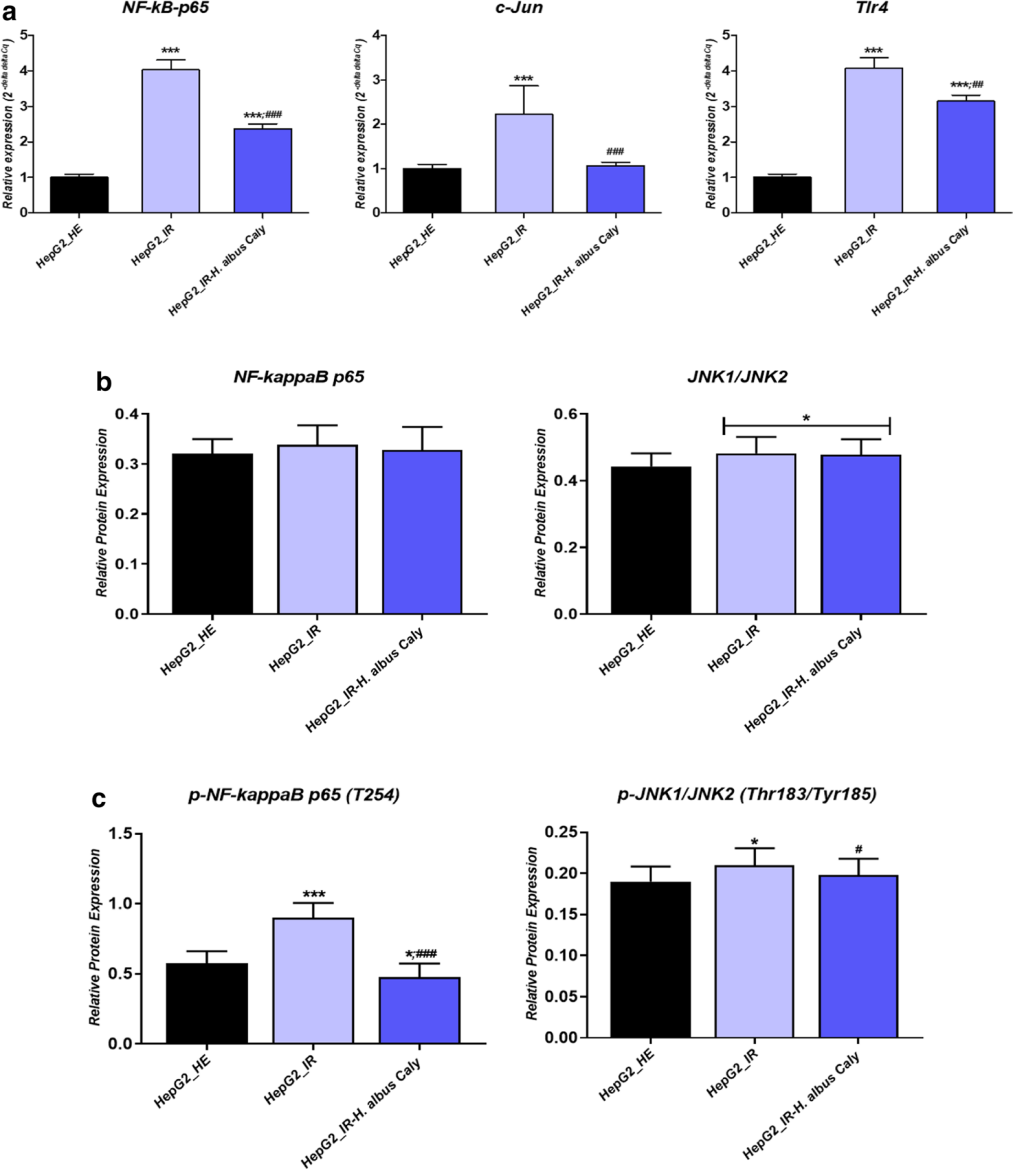

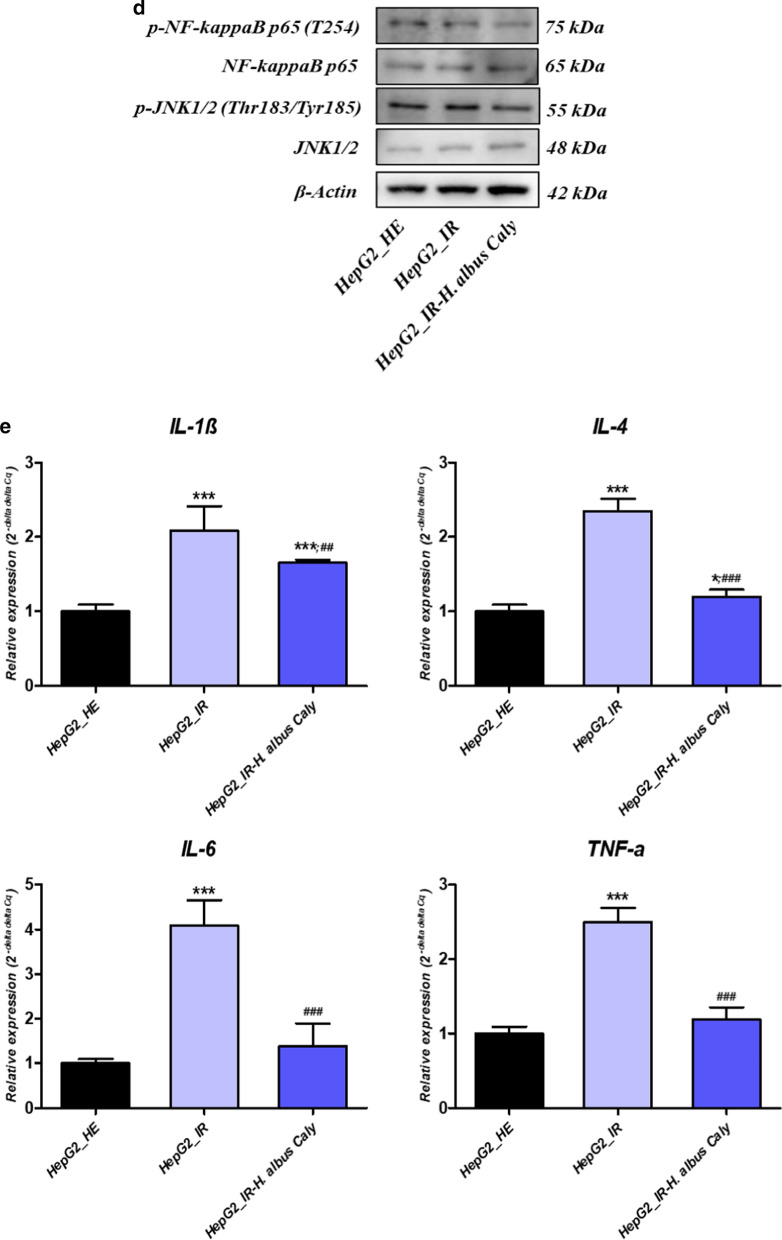
Effect of total calystegines on the *NF-κB/JNK/TLR4* inflammatory pathway triggered by insulin resistance condition in HepG2 cells. **a** Relative gene expression of c*-Jun*, *NF-κB* and *Tlr4* factors. **b** Quantitative representation of total NF- κB p65 and JNK1/2 proteins. **c** Quantitative representation of phosphorylated NF- κB p65(T254) and JNK(Thr183/Tyr185) proteins. **d** Representative chemiluminescent blots for analysed proteins. **e** Relative levels of main pro-inflammatory cytokines transcripts. Representative data from three independent experiments are shown ± SD (n = 3). An asterisk (*) indicates a comparison of IR group to untreated healthy cells. A hashtag (#) indicates a comparison of IR group pre-treated with calystegines to IR untreated healthy cells. */#*p* < 0.05, **/##*p* < 0.01, ***/###*p* < 0.001. HepG2_HE: HepG2 healthy untreated cells; HepG2_IR: Insulin resistant HepG2 cells exposed to high concentrations of insulin and glucose. HepG2_IR-*H. albus*_Caly: Insulin resistant HepG2 cells exposed to high concentrations of insulin and glucose and pre-treated with 250 μg/ml calystegines extracted from *Hyoscyamus albus* seeds

Since glucotoxicity together with hyperinsulinemia are believed to among others activate the NF-κB transcription factor and play a critical role in the induction of proinflammatory gene expression, that strongly promote the extension of the inflammatory process in liver, treated and untreated HepG2 cells were subjected to proinflammatory activation analysis. Insulin resistant cells displayed elevated levels of *c-Jun*, *NF-κB* and *Tlr4* mRNA in opposition to healthy cells, suggesting the initiation of an inflammatory response to high concentrations of glucose and insulin (Fig. 9a). Moreover, immunoblotting outcomes revealed an increase in the phosphorylation of NF- κB p65 at T254, and JNK at Thr183/Tyr185 (Fig. 9b) following HI/HG induction, while no obvious elevation in total p65 and total JNK proteins has been recorded under the same experimental conditions (Fig. 9b). In a remarkable manner, calystegines from *Hyoscyamus albus* lowered the expression of all the *c-Jun*, *NF-κB* and *Tlr4* transcripts, as well as the activation of *NF-κB* and JNK proteins through the inhibition of their phosphorylation, in comparison to IR untreated cells (*p* < 0.001). Moreover, calystegines pre-treatment resulted in a diminished p- NF- κB(T254)/p65 and p-JNK(Thr183/Tyr185)/JNK1/2 ratios when compared to both control groups (*p* < 0.05). As a consequence of NF-*κ*B pathway activation, real time amplification showed an overexpression of the main pro-inflammatory cytokines found in liver tissue, namely *Il-1β*, *Il-4*, *Il-6* and *TNF-α* in a significant manner by contrast to healthy group of cells (*p* < 0.001); while pre-treatment of cells with nortropane alkaloids mixture prevented the excessive transcription of the same genes (Fig. 9e), and even normalized the expression of *Il-6* and *TNF-α* mRNA when compared to healthy cultures; hinting the fact that calystegines may reduce glucotoxicity-triggered inflammatory responses through the suppression of the *NF-κB/JNK/TLR4* axis and the downstream proinflammatory cytokines recruitment.

## Discussion

In recent years, prevalence of obesity, diabetes and other metabolic disorders has rapidly increased highlighting the need to develop more appropriate therapies due to the heterogenic nature of these illnesses. Pharmacological hypoglycaemic drugs like insulin and metformin are still the first-choice option for affected individuals. However, in accordance to latest findings, some of the herbal derivatives can be applied as a dietary adjuvant supporting recovery of patients. For that reason, WHO recommends the studies of traditional plant treatments due to their relatively low cost, no-side effects and effectiveness as an anti-diabetic agents [30]. Since now, several plant species have been tested against insulin resistance revealing the agents responsible for therapeutic effects including glycosides, saponins, alkaloids and flavonoids.

In the presented study we have investigated the effects of calystegines from *Hyoscyamus albus* on HepG2 cells in which hyperinsulinemia and glucotoxicity was induced by the treatment with insulin and glucose. This is the first study showing insulin sensitising effects of this extract on cells of hepatic origin.

Results from cytotoxicity testing revealed that, calystegines showed no toxicity in all of the tested doses contributing to a regular cell cycle preservation. Based on screening assays, most potent concentration of extracts was selected to further testing with HepG2 cells treated with high concentrations of insulin and glucose as a model of insulin resistance. Calystegines augmented impaired proliferation potential of insulin resistant cells as evidenced by Tox8 assay. Previous studies, including our own, clearly indicated that proliferation rate and viability of cells is strongly affected by insulin resistance and that enhancement of insulin sensitivity is able to restore the progression of cell cycle [31–33].

To further evaluate the role of *Hyoscyamus albus’s* calystegines on cell death in insulin resistant HepG2 cells, the Muse® Annexin V & Dead Cell flow cytometric assay was performed. The obtained results clearly indicated that calystegines promote cell survival while reducing apoptosis. To further investigate the viability of cells, expression of genes involved in the apoptosis was investigated with RT-PCR. High abundance of p53, p21 and BAX while reduced Bcl-2 mRNA levels were characterised for insulin resistant HepG2. However, treatment of cells with calystegines from *Hyoscyamus albus* reversed the expression pattern of these genes clearly indicating that studied substances promote cell survival and reduce insulin resistance-related apoptosis. Obtained findings are especially important due to the fact that prolonged insulin treatment prime apoptosis and cell death-inducing mechanisms as a result of oxidative stress in β-cells [34]. Progressive insulin resistance is also directly associated with hepatocyte apoptosis due to the caspase activation. For that reason, in the next step of the experiment the presence of multiple caspases (caspase-1, 3, 4, 5, 6, 7, 8, and 9) in cells was established with flow cytometry-based Muse™ MultiCaspase Assay and RT-PCR analysis of caspase-3 and caspase-9 expression. In accordance to recent findings, caspase-2 promotes obesity, the metabolic syndrome and non-alcoholic fatty liver disease [35] and for that reason may become a potential target to correct obesity and its associated comorbidities. Similar results were found for caspase-3 as its inactivation protects against hepatic cell death and ameliorates fibrogenesis in a diet-induced NASH model [36]. However, no specific inhibitor of that proteins has been developed yet. Interestingly, in the presented study we have confirmed that caspase activation triggers apoptosis in insulin resistant HepG2 and that calystegines from *Hyoscyamus albus* are able to reverse that phenomenon. Blocking caspases activity with tested extract may represent a novel approach for the reestablishment of cellular homeostasis and exert the therapeutic outcome.

High circulating levels of glucose induce generation of reactive oxygen species (ROS) which worsen insulin resistant state and drive progressive inflammation [37]. It was shown, that generation of mitochondrial ROS strongly contributes to hepatic insulin resistance. ROS impair insulin signalling pathway, activate JNK and deteriorate phosphorylation of IRS contributing to the development of hepatic insulin resistance. For that reason, both ROS and mitochondria are now recognised as a therapeutic target against metabolic disorders. Interestingly, we have found that tested calystegines reversed overexpression of glutathione peroxidase (GPX) and Catalase (CAT); moreover, nortropanes efficiently restored the lost superoxide dismutase 1 (*Sod1)* and superoxide dismutase 2 (*Sod2)* expression, suggesting that treated cells were characterised by oxidative homeostasis and reduced ROS as increased expression of antioxidative enzymes serves as a protective mechanism. It is expected from drug candidates to enhance mitochondrial function while reducing ROS accumulation in cells as a strategy to restore proper insulin signalling and glucose uptake. Herein we showed, that *Hyoscyamus albus* extracts significantly reduced glucose/insulin-induced ROS accumulation and reduce mitochondrial removal from cells. It is worth noting, that calystegines supported mitochondrial fusion which enables to compensate metabolic defects of these organelles.

Defects in insulin signalling pathway which leads to disruption of glucose homeostasis are believed to be key players in the development of insulin resistance [38]. Activation of INSR triggers a cascade of events including IRS-1 tyrosine phosphorylation, increased PI3-kinase activity, activation of AKT and glucose transporters and ultimately leads to increased insulin-stimulated glucose disposal [39]. Obtained results revealed that the insulin receptor expression was reduced, producing an insulin-resistant state in HepG2 cells treated with high concentration of glucose and insulin. The expression of other genes involved in the pathway like IRS, AKT and PI3K was also significantly decreased. After treatment of cells with the calystegines from *Hyoscyamus albus* expression of these genes was significantly enhanced. In support to that, increased glucose uptake was observed on the basis of 2-NBDG staining. We have also showed that calystegines from *Hyoscyamus albus* significantly decreased in overexpression of glucose 6-phosphatase catalytic subunit (G6PC) and Foxo1, which regulates hepatic gluconeogenesis, in insulin resistant cells. Depletion of liver specific G6PC protects from diabetes in L-G6pc^−/−^ mice fed a high fat/high sucrose diet [40]. On the other hand, FOXO1 overexpression was found in patients with steatohepatitis and hepatic insulin resistance [41]. It appeared that, tested calystegines improve insulin sensitivity via modulation of key players in the insulin signalling pathway and liver specific glucogenesis. The beneficial effects of *Hyoscyamus albus* extracts against insulin resistance observed in the presented study may result from the activation of PI3K/AKT pathway. That molecular axis is significantly involved in regulation of insulin signalling and glucose metabolism. AKT promotes translocation of glucose transporter type 4 (GLUT-4) and increase glucose uptake in mice fed on high fat diet [42].

Furthermore, we investigated whether tested calystegines modulate the expression of SIRT1 transcription factors which is associated with glucose and lipid metabolism, however its role in the development of insulin resistance and diabetes is still not fully elucidated [43]. It was showed, that loss of SIRT1 triggers DNA damage [44] and calcification in a diabetic environment [45]. A well-known SIRT1 activator—resveratrol (RES)—was shown to attenuate diabetes [46] while SIRT1 knockdown leads to insulin resistance, enhanced cholesterol and free fatty acid levels [47]. It has been shown, that palmitate treated insulinoma cell line clone 1E (INS-1E) displayed decreased SIRT1 levels, however, RES enhanced it expression as well as the genes associated with mitochondrial biogenesis and lipid metabolism providing the evidence that SIRT1 activation mitigates diabetes [22]. Here we have shown, that calystegines from *Hyoscyamus albus* similarly to RES are able to activate SIRT1 in glucose/insulin treated cells and in consequence enhance insulin sensitivity and cellular viability. The results of present study indicate that calystegines act via SIRT1 in PA-induced HepG2 cells providing an insight into the mechanism by which these substances may prevent diabetes and supporting their application as a clinical intervention.

Multiple studies have indicated that chronic inflammation is an important part of the clinical picture of obesity-induced insulin resistance patients [48]. Adipose tissue of obese individuals is abundantly infiltrated in adipose tissue macrophages (ATMs) with M1 phenotype secreting a set of pro-inflammatory cytokines including tumor necrosis factor-α (TNF-α), interleukin-1β (IL-1β) which worsens the insulin resistant state. Inflammatory factors are also able to activate the c-Jun N-terminal kinase (JNK) family which in turns triggers activation of nuclear factor-κB (NF-κB) and following events leading to the enhanced secretion of proinflammatory cytokines and aggravation of insulin resistance. The profound role of NF-κB in the development of insulin resistance was proved by Zeng et al. [49] who discovered that the hepatic NF-κB pathway plays a pivotal role in diet-induced insulin resistance and that silencing of NF-κB using small interfering RNA protects against disease development. Additionally, NF-κB can be activated by tools like receptor 4 (TLR4) which is also associated with diabetes development being overexpressed in various cell types and organs in affected individuals [50–52]. For that reason, searching for antidiabetic medications or lifestyle intervention able to reduce inflammation and in turn improve insulin resistance is fully justified. Herein we have shown, that calystegines from *Hyoscyamus albus* significantly reversed overexpression of NF-κB, JNK and TLR4 in insulin resistant HepG2 cells. Furthermore, we noted significantly decreased expression of pro-inflammatory factors like IL-1β, TNF-α, interleukin 4 (IL-4) and interleukin 6 (IL-6). Observed effects correlates with anti-inflammatory action of antidiabetic agents like metformin [53] and dipeptidyl peptidase (DPP)-4 inhibitors [54]. It was shown that inhibition of TLR 4 prevents development of autoimmune diabetes [55] while inhibition of IκB kinase β (IKKβ)/ NF-κB attenuates high-fat diet–induced obesity and glucose intolerance in mice [56].

## Conclusion

To summarize, our results demonstrate that calystegines from *Hyoscyamus albus* provide cytoprotection to the HepG2 cells against insulin/glucose induced insulin resistance and apoptosis due to activation of PI3K/AKT and SIRT1/NF-kB/JNK signalling pathways. The current study highlights the utility of *Hyoscyamus albus* as an anti-diabetic agent and supports the need for future in vivo studies.

## Data Availability

The data that support the findings of this study are available from the corresponding author, upon reasonable request.

## Abbreviations

2-NBDG: 2-[N-(7-nitrobenz-2-oxa-1,3-diazol-4-yl) amino]-2-deoxy-D-glucose
7-AAD: 7-Aminoactinomycin D
Akt1: Serine/threonine 308 Kinase 1
Akt2: Serine/threonine kinase 2
ATCC: American Type Culture Collection
ATMs: Protein kinase ataxia-telangiectasia mutated
Bax: BCl-2 associated X protein
BCA: Bicinchoninic acid assay
Bcl-2: B-cell lymphoma 2
BSA: Bovine serum albumin
cAMP: Cyclic adenosine monophosphate
CAP: Catabolite activator protein
Casp3: Caspase 3
Casp9: Caspase 9
CAT: Catalase
CCR2: C–C Motif Chemokine Receptor 2
c-Jun: Jun Proto-Oncogene, AP-1 Transcription Factor Subunit
DAPI: 4′,6-Diamidino-2-phenylindole
DMEM: Dulbecco's Modified Eagle Medium
EDTA: Disodium Ethylenediaminetetraacetic acid
FBS: Fetal bovine serum
Foxo1: Forkhead box O1
G6PC: Glucose 6 phosphate Catalytic subunit
GADPH: Glyceraldehyde-3-phosphate dehydrogenase.
GLUT-4: Glucose transporter 4
GPx: Glutathione Peroxidase
HBSS: Hanks' Balanced Salt Solution
HepG2: Hepatoma G2
HG: Hyperglycemia
HI: Hyperinsulinemia
IKK: IκB kinase
IL-1β: Interleukin 1-beta
IL-4: Interleukin 4
IL-6: Interleukin 6
IL-8: Interleukin 8
INS-1E: Cellosaurus cell line
Insr: Insulin Receptor
Irs2: Insulin Receptor Substrate 2
JNK: C-Jun N-terminal kinase
JNK1/JNK2(phospho-Thr183/Tyr185): Phosphorylated c-Jun N-terminal kinase.
MCP-1: Monocyte chemoattractant protein 1
Mfn1: Mitofusin 1
Mfn2: Mitofusin 2
NAFLD: Non-alcoholic fatty liver disease
NASH: Non-alcoholic steatohepatitis
NF-kappaB p65(phospho-T254): Phosphorylated Nuclear Factor-kappa-B transcription factor p65
NF-kB-p65: Nuclear Factor-kappa-B transcription factor p65
NF-κB: Nuclear factor kappa B
NO: Nitric oxide
NORD: National Organization of Rare Disease
p21: Cyclin-dependent kinase inhibitor 1
P53: Tumor suppressor p53
Parkin: Parkin RBR E3 ubiquitin protein ligase (PARK2)
PBS: Phosphate-buffered saline
PGC-1α: Peroxisome proliferator-activated receptor-gamma coactivator
Pi3k: Phosphoinositide 3-Kinase
Pink1: PTEN-induced putative kinase 1
PKA: Protein kinase A
PVDF: Polyvinylidene fluoride
RES: Resveratrol
ROS: Reactive oxygen species
Sirt1: Sirtuin 1
SOD: Superoxide dismutase
Sod1 (Cu/Zn SOD): Copper-zinc-dependant superoxide dismutase (CuZnSOD
Sod2 (Mn SOD): Manganese-dependent superoxide dismutase (MnSOD)
TBST: Tris-buffered saline, 0.1% Tween 20
TLR: Toll like receptor
TLR4: Toll like receptor 4
TNF-α: Tumor Necrosis Factor alpha
TOR/wTOR: Target of rapamycin/Mammalian Target of rapamycin
WHO: World health organization
