# Correction: *Hyoscyamus albus* nortropane alkaloids reduce hyperglycemia and hyperinsulinemia induced in HepG2 cells through the regulation of *SIRT1*/*NF-kB/JNK* pathway

**DOI:** 10.1186/s12964-023-01056-w

**Published:** 2023-01-20

**Authors:** Anna Kowalczuk, Nabila Bourebaba, Katarzyna Kornicka-Garbowska, Eliza Turlej, Krzysztof Marycz, Lynda Bourebaba

**Affiliations:** 1 National Medicines Institute, Chełmska 30/34, 00-725 Warsaw, Poland; 2 International Institute of Translational Medicine, Jesionowa 11, 55-114 Malin, Wisznia Mała Poland; 3 Department of Experimental Biology, Faculty of Biology and Animal Science, Wrocław University of Environmental and Life Sciences, Norwida 27B, 50-375 Wrocław, Poland; 4 Collegium Medicum, Institute of Medical Science, Cardinal Stefan Wyszyński University (UKSW), Dewajtis 5, 01-815 Warsaw, Poland

**Correction to** **:** **Cell Commun Signal (2021) 19:61**

**https://doi.org/10.1186/s12964-021-00735-w**

Following publication of the original article [1], the authors reported an error in the funding section. The research grant “Inhibition of tyrosine phosphatase as a strategy to enhance insulin sensitivity through activation of chaperone mediated autophagy and amelioration of inflammation and cellular stress in the liver of equine metabolic syndrome (EMS) horses” (Grant No. 2018/29/B/NZ7/02662), financed by The National Science Centre in Poland is removed from the Acknowledgments/Financial disclosure.

The authors would like to apologise for any inconvenience caused.
